# A Comprehensive Survey on the Terpene Synthase Gene Family Provides New Insight into Its Evolutionary Patterns

**DOI:** 10.1093/gbe/evz142

**Published:** 2019-07-15

**Authors:** Shu-Ye Jiang, Jingjing Jin, Rajani Sarojam, Srinivasan Ramachandran

**Affiliations:** 1 Genome Structural Biology Group, Temasek Life Sciences Laboratory, National University of Singapore, Singapore; 2 School of Computing, National University of Singapore, Singapore; 3 China Tobacco Gene Research Centre, Zhengzhou Tobacco Research Institute of CNTC, Zhengzhou, China

**Keywords:** terpene synthase, evolution, genome RNA-Seq, isoprenyl diphosphate synthase

## Abstract

Terpenes are organic compounds and play important roles in plant growth and development as well as in mediating interactions of plants with the environment. Terpene synthases (*TPS*s) are the key enzymes responsible for the biosynthesis of terpenes. Although some species were employed for the genome-wide identification and characterization of the *TPS* family, limited information is available regarding the evolution, expansion, and retention mechanisms occurring in this gene family. We performed a genome-wide identification of the *TPS* family members in 50 sequenced genomes. Additionally, we also characterized the *TPS* family from aromatic spearmint and basil plants using RNA-Seq data. No *TPS*s were identified in algae genomes but the remaining plant species encoded various numbers of the family members ranging from 2 to 79 full-length *TPS*s. Some species showed lineage-specific expansion of certain subfamilies, which might have contributed toward species or ecotype divergence or environmental adaptation. A large-scale family expansion was observed mainly in dicot and monocot plants, which was accompanied by frequent domain loss. Both tandem and segmental duplication significantly contributed toward family expansion and expression divergence and played important roles in the survival of these expanded genes. Our data provide new insight into the *TPS* family expansion and evolution and suggest that *TPS*s might have originated from isoprenyl diphosphate synthase genes.

## Introduction

Terpenes are the largest and most structurally diverse class of natural compounds (Tholl 2015). They are generally produced by plants, fungi, bacteria, or a few of insects. To date, around 50,000 terpenoid metabolites, including monoterpenes, sesquiterpenes, and diterpenes, have been identified from higher plants, liverworts, and fungi (Yamada et al. 2015). The universal precursor for all types of terpenes are isopentenyl diphosphate (IPP) and dimethylallyl diphosphate (DMAPP), which are produced from mevalonate pathway (MVA) in cytoplasm or by the 2-*C*-methyl-d-erythritol 4-phosphate (MEP) pathway in plastids (Vranová et al. 2013). Under the action of prenyltransferases, DMAPPs are fused with various numbers of IPP units to synthesize geranyl diphosphate (GPP, C10), farnesyl diphosphate (FPP, C15), and geranylgeranyl diphosphate (GGPP, C20). Terpene synthases (TPSs) are a diverse class of enzymes which catalyses the biosynthesis of hemiterpenes (C5), monoterpenes (C10), sesquiterpenes (C15), or diterpenes (C20) using the substrates DMAPP, GPP, FPP, or GGPP, respectively (McGarvey and Croteau 1995). Few TPSs are involved in synthesis of primary terpene like gibberellins but majority participate in the production of specialized secondary metabolite involved in plant ecological interactions. Each full-length TPS is characterized by two conserved domains with Pfam (Finn et al. 2016) ID PF01397 (N-terminal) and PF03936 (C-terminal) (Starks et al. 1997). The N-terminal domain has a conserved RRX_8_W (R, arginine, W, tryptophan and X, alternative amino acid) motif and the C-terminal domain contains two highly conserved aspartate-rich motifs. One of them is the DDxxD motif, which is involved in the coordination of divalent ion(s), water molecules and the stabilization of the active site (Starks et al. 1997; Rynkiewicz et al. 2001; Whittington et al. 2002). The second motif in the C-terminal domain is the NSE/DTE motif. These two motifs flank the entrance of the active site and function in binding a trinuclear magnesium cluster (Christianson 2006; Degenhardt et al. 2009). Although TPSs contain two conserved domains, most phylogenetic analysis has been carried out using the full-length amino acid sequences. Thus, the resulting phylogenetic tree may be affected by nonconserved regions and may not represent their true evolutionary relationships.

The *TPS* family has been split into six groups/subfamilies according to phylogenetic analysis namely *TPS-a*, *-b*, *-c*, *-d*, *-e*, and *-f* (Bohlmann et al. 1997). This classification was based on full-length amino acids and the chosen TPSs were either from angiosperms or gymnosperms. Later, both *TPS-e* and *TPS-f* were merged into one group/subfamily designated as *TPS-e/f* since *TPS-f* was derived from *TPS-e* and they were clustered into one clade (Chen et al. 2011). Further, two more subfamilies were identified, *TPS-g* and *TPS-h* (Chen et al. 2011). The former is found in angiosperms and the latter only in *Selaginella moellendorffii* (Chen et al. 2011). Thus, based on phylogenetic analysis using full-length amino acid sequences, a total of seven subfamilies have been clustered including *TPS-a*, *TPS-b*, *TPS-c*, *TPS-d*, *TPS-e/f*, *TPS-g*, and *TPS-h*. The *TPS-a* subfamily encodes only sesqui-TPSs that are found in both dicot and monocot plants. In this subfamily, the secondary “R” (arginine) of the arginine/tryptophan motif RRX_8_W is not conserved (Martin et al. 2010). In contrast, the angiosperm-specific *TPS-b* subfamily encodes monoTPSs and contains the conserved R(R)X_8_W motif, which functions in the initiation of the isomerization cyclization reaction (Williams et al. 1998) or in stabilizing the protein through electrostatic interactions (Hyatt et al. 2007). The *TPS-g* subfamily is another angiosperm mono-TPS subfamily, encoding TPSs without the R(R)X_8_W motif. These TPSs are required for biosynthesis of acyclic monoterpenes, which form floral VOCs (Dudareva et al. 2013). The *TPS-c* subfamily is present in land plants and is characterized by the “DXDD” motif but not the “DDXXD” motif in their proteins, which was detected in other subfamilies (Chen et al. 2011). The *TPS-d* subfamily members may encode mono-, sesqui-, and di-TPSs and they are gymnosperm-specific. The *TPS-e/f* subfamily is mainly detected in vascular plants and they encode copalyl diphosphate synthases and kaurene synthases, responsible for gibberellic acid biosynthesis. The *TPS-g* subfamily is closely related to *TPS-b* but lacks the conserved R(R)X_8_W motif in its encoded proteins and its members may function in producing acyclic mono-, sesqui-, and diterpene products (Chen et al. 2011). The *TPS-h* subfamily is found only in *S. moellendorffii* and its members encode both “DXDD” and “DDXXD” motifs (Chen et al. 2011).

With the availability of whole genome sequences, the genome-wide identification of *TPS*s has been carried out in several species. The Arabidopsis genome, which is the first plant genome to be sequenced (The Arabidopsis Genome Initiative 2000), encodes 32 full-length *TPS*s including 22 *TPS-a*, 6 *TPS-b*, 1 *TPS-c*, 2 *TPS-e/f*, and 1 *TPS-g* (Aubourg et al. 2002). In grapevine (*Vitis vinifera*), 69 putatively functional *TPS*s have been identified by hidden Markov model (HMM) searches (Martin et al. 2010). In *Oryza sativa*, *Sorghum bicolor*, *Populus trichocarpa*, and *S.moellendorffii*, 34, 24, 32, and 14 *TPS*s have been identified, respectively (Chen et al. 2011). In tomato (*Solanum lycopersicum*), 44 members were identified by TBLASTN searches (Falara et al. 2011). In *Physcomitrella patens*, only one *TPS* was identified (Li et al. 2012). The site-directed mutagenesis and knock-out mutant analysis of this TPS revealed the functions of two conserved motifs (DVDD and DDYFD) and their roles in the biosynthesis of diterpenes (Hayashi et al. 2006; Zhan et al. 2015). Both *Eucalyptus grandis* and *Eucalyptus globulus* encode 113 and 106 *TPS*s, respectively by BLAST searches using sequences from conserved regions (Külheim et al. 2015). These data show that different genomes encode various numbers of *TPS*s with different family expansion mechanisms. Data also showed the difference in the evolutionary patterns between monocots and dicots (Chen et al. 2011). Generally, compared with the numbers of species whose whole genomes have been sequenced, limited species have been employed for the genome-wide identification and characterization of the *TPS* family. On the other hand, the classification of the *TPS* family in each species was carried out by using only the full-length amino acid sequences. One may argue whether the classification system can be used for the majority of plant species and whether using the full-length amino acid sequences but not domain sequences is the best way to evaluate the phylogenetic relationship.

In this study, we carried out genome-wide identification and characterization of all *TPS* genes encoded by 50 lower and higher plant species including algae, liverwort, moss, gymnosperm, and angiosperm. We only identified these TPSs that contain either PF01397 or PF03936 or both of them. Other TPSs such as oxidosqualene cyclase and squalene-hopene cyclase were excluded due to their lack in these two domains. We then examined their transcript profiling by microarray and RNA-Seq data sets. We also surveyed their expression divergence among various tissues to further annotate their biological functions and to explore the potential retention mechanisms. Our data show that the *TPS* gene family is not ubiquitous in the plant kingdom as no member was found in all of the examined species of green algae. Plant species have evolved different sizes of *TPS* families and their subfamilies to synthesize a specific set of terpene compounds to interact with different biotic/abiotic environments. Both tandem and segmental duplication were regarded as the main mechanisms driving *TPS* expansion. Our data also showed that both monocot and dicot *TPS* genes exhibit different evolutionary and expansion histories. Some subfamilies of *TPS*s were expanded in specific species, especially in some trees and grasses, which might have played a role in species diversification.

## Materials and Methods

### Databases for Genomic, cDNA, and Protein Sequences

In this study, genomic, cDNA, and protein sequences from 50 species were used for genome-wide identification of *TPS*s and related analysis. Their genomes from all of these species have been completely sequenced and annotated. These sequence data for most of them were downloaded from the release v11 of Phytozome database (http://phytozome.jgi.doe.gov/; last accessed July 16, 2019) or the NCBI genome database (https://www.ncbi.nlm.nih.gov/genome; last accessed July 16, 2019). The sequences for the species *Pic.abies* were downloaded from the website http://congenie.org/; last accessed July 16, 2019 (Nystedt et al. 2013). For both spearmint (short name mint, *Mentha spicata*) and sweet basil (*Ocimum basilicum*), only assembled cDNA from RNA-Seq and their deduced amino acid sequences were used for this study. For another basil species *Ocimumtenuiflorum*, its genomic, cDNA, and protein sequences were downloaded from the TulsiDb (Upadhyay et al. 2015; http://caps.ncbs.res.in/Ote/; last accessed July 16, 2019).

### Genome-Wide Identification of *TPS*s by Profile HMM and BLASTP Searches

All identified TPSs contain a conserved domain structure with the Pfam (pfam.xfam.org/) ID PF01397 or PF03936 and many of TPSs contain both of these domains. Representative domain sequences for either PF01397 or PF03936 were downloaded from the Pfam database and were used for sequence alignment using the program Clustal X 2.0 (Thompson et al. 1997; http://www.clustal.org/; last accessed July 16, 2019). The aligned domain sequences were used to construct HMM profiles for either PF01397 or PF03936 with the HMMER 3.0 (http://hmmer.org/; last accessed July 16, 2019). We used these two profiles to carry out HMM searches against the above mentioned 50 protein databases with E-value cut-off of 1.0. Similarly, the HMM searches were also used to identify all putative TPSs in both mint and basil by searching against corresponding protein sequences deduced from assembled unigenes. These searches resulted in two sets of sequences containing either PF01397 or PF03936 domains. We then manually inspected these sequences by confirming the presence of these domains. Any artifacts were removed, which were lack either PF01397 or PF03936. The full-length TPSs with two domains were collected by combining these two sets of resulted data. On the other hand, to minimize the possible loss of any of putative TPSs, standalone BLASTP searches were also carried out by using the representative TPSs from the HMM searches with E-value cut at 0.01. After domain verification, these identified sequences were classified as the putative TPSs, which were used for all the remaining investigation in this study.

### Phylogenetic Tree Construction and *TPS* Classification

A typical full-length *TPS* usually encodes both PF01397 and PF03936 domains. To better understand the *TPS* family, these two domains were separately used for phylogenetic tree constructions. Domain sequences were identified by the HMM searches and were confirmed by the Pfam database. The achieved domain amino acid sequences were aligned using Clustal X 2.0 (Thompson et al. 1997; http://www.clustal.org/; last accessed July 16, 2019). The aligned sequences were used for phylogenetic tree construction by the MEGA7 program (Kumar, Stecher, et al. 2016). The trees were generated by Maximum Likelihood method based on the Jones–Taylor–Thornton (JTT) matrix-based model (Jones et al. 1992) and Bootstrap method was used for phylogeny test with 1,000 replications. The bootstrap values were added in all presented phylogenetic trees. The generated trees were confirmed by the Bayesian analyses using the MrBayes 3.2.6 program (Huelsenbeck and Ronquist 2001; http://mrbayes.csit.fsu.edu/; last accessed July 16, 2019). The family classification was carried out according to the system developed by Chen et al. (2011), where *TPS-e* and *TPS-f* were combined together into the subfamily *TPS-e/f*. We used either PF01397 or PF03936 for phylogenetic analysis followed by the family classification. The phylogenetic trees from these two domains showed the differences for some species. In this case, the trees deduced from the domain PF01397 were used for classification.

### Estimation of Nonsynonymous Substitutions per Site (*Ka*)/Synonymous Substitutions per Site (*Ks*) Ratios and Horizontally Transferred Genes

Expanded genes from duplication or transposition were subjected to various selective stresses. To estimate *Ka*, *Ks*, and their ratios of these genes, their protein domain sequences were aligned, which were subsequently converted into the original cDNA sequences by using the PAL2NAL program (Suyama et al. 2006). The aligned cDNA sequences were used to estimate *Ka*, *Ks*, and their ratios using the yn00 program of the PAML4b package (Yang and Nielsen 2000).

To determine purifying/positively selected amino acid sites in a *TPS* family, all the domain amino acid sequences were aligned using the Clustal X 2.0 program. The aligned amino acid sequences were used 1) for constructing phylogenetic trees and 2) as a guideline for corresponding cDNA sequence alignment. Both phylogenetic trees and aligned cDNA sequences were then subjected to the “sitewise likelihood-ratio” (SLR) program (Massingham and Goldman 2005) to detect corresponding amino acid sites with purifying/positive selection. The SLR program is specially designed to detect coding sequence sites under purifying or positive selection by analyzing their alignment of sequences (Massingham and Goldman 2005). The program combines the maximum likelihood phylogenetic approach (Nielsen and Yang 1998) with the site-wise statistical test (Suzuki and Gojobori 1999). The positively or negatively selected sites were identified by their *Ka*/*Ks* ratios with a confidence interval for each ratio given by a *P* value and an adjusted *P* value (Adj.Pval) from multiple comparisons (Massingham and Goldman 2005). We used both *P* value and Adj.Pval to evaluate the positively selected sites. We used Windows (v1.3) for our analysis, which was downloaded from the website http://www.ebi.ac.uk/goldman-srv/SLR/; last accessed July 16, 2019.

Horizontally transferred genes (HTGs) were identified between bacterium and plant genomes. Amino acid sequences from plant TPSs were used as queries to search against protein databases annotated from soil bacterium genome sequences using the standalone BLASTP program “ncbi-blast-2.4.0” (http://www.ncbi.nlm.nih.gov/books/NBK52637/; last accessed July 16, 2019). Candidate sequences, which showed a minimum 70% of queried protein coding regions aligned with an E-value cutoff level at 0.01, were selected to further investigation. A HTG from a plant to a soil bacterium genome was identified when a candidate sequence showed the high-sequence similarity (>70% coverage and >80% identities in amino acid sequence) in multiple plant species but no significant homolog in any other bacteria.

### Detection of Tandemly/Segmentally Duplicated and Transposon/Retrotransposon-Expanded *TPS*s

Tandemly duplicated genes were identified by their sequence similarity and chromosomal localization according to our previous description (Jiang et al. 2009). Briefly, a tandemly duplicated gene was identified by comparing with its putative parental gene. Firstly, the gene should contain at least 30% of its sequence, which is able to be aligned with the parental sequence by BLASTP searches with an E value cutoff level at 0.01. Secondary, they share at least 70% identity in their amino acid sequences. Thirdly, these two genes should be no more than ten genes apart and are located within 100 kb for genomes with <200 Mb in size and 350 kb for the remaining genomes.

These genes, which were located on segmentally duplicated chromosome blocks, were designated as segmentally duplicated genes. Segmentally duplicated blocks were identified by the DAGchainer program (Haas et al. 2004) using sequence fragments flanking 50 kb upstream and downstream of *TPS*s as well as the corresponding whole genome sequences. At least five gene pairs were used to define a block during running the program.

To survey the contribution of mobile elements to the expansion of *TPS*s, the sequence fragments flanking 50 kb upstream and downstream of TPSs were also subjected to the identification of transposon-related elements including LTR-retrotransposon, retrogene, mutator-like transposable element (MULE), *hobo/Ac/Tam3* (*hAT*), *CACTA*, and *Helitron*. The LTR_ FINDER program (Xu and Wang 2007) was used to identify the full-length retrotransposons. Retrogenes were identified according to the criteria as described by Wang et al. (2006). The remaining mobile elements were identified according to our previous description (Jiang et al. 2009).

### Plant Materials and Growth Conditions

Both basil and mint plants were grown in greenhouse under natural light and temperature conditions. For mint, 1–2 cm leaves and for basil, 3–4 cm leaves were used for peltate glandular trichomes (PGT) isolation as described in Jin et al. (2014). Same size leaves were brushed to remove PGTs and checked under dissection microscope. Roots were harvested from 6- to 8-week-old plants.

### RNA Isolation, Sequencing, and Assembly

Collected samples from roots, leaves, leaf-PGT, and PGT tissues were used for total RNA preparation using the Spectrum Plant total RNA kit from Sigma according to the manufacturer’s instructions. After checking the ratio of OD260 to OD280 and the integrity by measuring the RNA Integrity Number (RIN) using Agilent 2100 bioanalyser, eligible RNA samples were subjected to the RNA library construction using the TruSeq RNA Sample Preparation Kits v2, set A (RS-122-2001, Illumina Inc.). The libraries were subjected to RNA-Seq by Hiseq-2000 (illumine Inc.). The unigene assembly and related bioinformatics analysis were carried out as described by Jin et al. (2014). The raw RNA-Seq data have been deposited into the DDBJ database (http://trace.ddbj.nig.ac.jp/dra/index_e.html; last accessed July 16, 2019) with accession numbers DRA001856.

### Expression Profiling of *TPS*s

Several expression data sets from either microarray or RNA-Seq experiments were achieved for profiling transcriptome of *TPS*s. For the wheat *TPS* expression analysis, the RNA-Seq data sets including 57 sets of running were downloaded from the European Nucleotide Archive database (http://www.ebi.ac.uk/ena; last accessed July 16, 2019) with accession number ERP004714. The data set was used to evaluate the expression patterns of the wheat *TPS*s and their expression divergence of tandemly or segmentally duplicated genes. For the Arabidopsis *TPS* expression analysis, the microarray data set containing 24 experimental samples were downloaded from the NCBI GEO data set (Barrett 2013; http://www.ncbi.nlm.nih.gov/geo/; last accessed July 16, 2019) with accession number GSE5634. The data set was used to investigate the Arabidopsis *TPS* expression patterns and their expression divergence after duplication. For the mint and basil *TPS* expression, a total of eight and four RNA samples were submitted to RNA-Seq analysis, respectively. The original data sets have been deposited into the DDBJ database as mentioned earlier. For RNA-Seq data, transcript abundance was estimated by raw read counts and FPKM (Fragments Per Kilobase of transcript per Million mapped reads) values, which were calculated from the three replicates for wheat and one replicate was carried out for mint and basil. The resulted FPKM values were log_2_-transformed and were then used to generate heat maps. For microarray data set, normalized expression values were calculated from raw data using the Agilent GeneSpring GX 11.5 software, which were used to construct heat map. The heat map was prepared by the Java Treeview (Saldanha 2004; http://jtreeview.sourceforge.net/; last accessed July 16, 2019). A tissue-preferred gene or expression divergence among expanded/duplicated genes was determined according to their tissue specificity and/or expression abundance among different tissues or under different abiotic/biotic stresses. Genes with at least two times difference in their log_2_-transformed or normalized expression value between tissues/treatments were submitted for statistical analysis (Student’s *t*-test). These genes which showed at least two times higher in one tissue when compared with any other tissues with a statistical difference at *P* < 0.05 were regarded as a tissue-preferred gene. Similarly, expression divergence of two genes was statistically determined when a gene showed at least two times difference in their expression abundance in any tissues or under any treatments.

## Results

### Different Plant Genomes Encode Highly Variable Sizes of the *TPS* Families

**Fig. 1.**
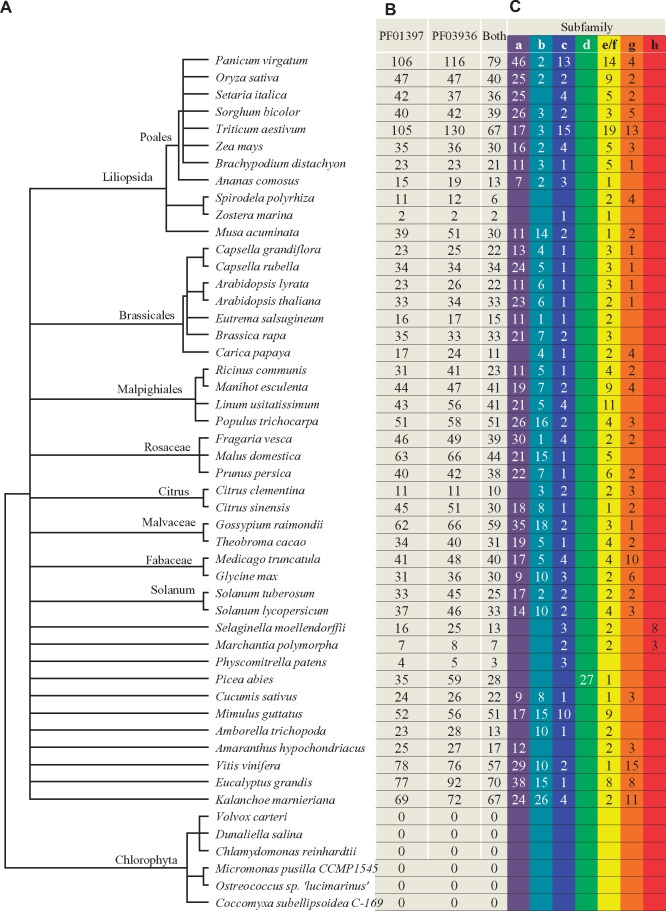
—Genome-wide identification of *TPS*s in 50 species. (*A*) Common tree of a total of 50 species used in this study. (*B*) Numbers of identified *TPS*s in these 50 species. We constructed the HMM profiling separately for each domain (PF01397 or PF03936) for the genome-wide identification of TPSs in each species. (*C*) Classification of subfamilies. Only those *TPS*s containing both PF01397 and PF03936 were subjected to classification.

To obtain a general profile of the *TPS* family in the plant kingdom, the assembled genome sequences and their annotated protein sequences from all of the 50 species were downloaded for the HMM searches to figure out all members encoding either PF01397 or PF03936 domains (see Materials and Methods). These species include algae, liverwort, moss, gymnosperm, and angiosperm (fig. 1*A*). Our data showed that no *TPS* was detected from all six genomes of Chlorophyta (fig. 1*B*). Besides green algae, we searched 12 other algal genomes including *Porphyridium purpureum* (Bhattacharya et al. 2013) (http://cyanophora.rutgers.edu/porphyridium/; last accessed July 16, 2019), *Cyanidioschyzon merolae* (Matsuzaki et al. 2004) (http://merolae.biol.s.u-tokyo.ac.jp/; last accessed July 16, 2019), *Bigelowiella natans* (Curtis et al. 2012), *Ectocarpus siliculosus* (Cock et al. 2010), *Galdieria sulphuraria*, *Gracilaria chilensis*, *Gracilariopsis lemaneiformis*, *Guillardia theta*, *Phaeodactylum tricornutum*, *Porphyra pulchra*, *Thalassiosira oceanica*, and *Thalassiosira pseudonana*, whose whole genome sequences were available from the NCBI database (http://www.ncbi.nlm.nih.gov/genome/; last accessed July 16, 2019). However, no *TPS* was detected from all these genomes.

For the remaining 44 non-alga plant species, variable sizes of family members were observed (fig. 1*B* andsupplementary table S1 _Sheet1, Sheet_2, and _Sheet3, Supplementary Material online). Some of these members contained only either PF01397 or PF03936 domain and the remaining had both of them, which were designated as full-length TPSs. Species from liverwort, moss, and aquatic plants (*Zostera marina*, *P.patens*, and *Spirodela polyrhiza*) mostly encoded small size of *TPS* families ranging from two to six full-length *TPS*s. The remaining species encoded at least 11 full-length *TPS*s. Only two TPSs Zosma26g01520 and Zosma52g00660 were identified in the aquatic plants (fig. 1) and they possessed both domains. In dicot plants, the species *E. grandis* and *Aquilegia coerulea* encoded the highest numbers (70) of full-length TPSs. In monocot plants, the highest numbers (79) of full-length TPSs were detected in the species *Panicum virgatum* (fig. 1).

### Classification of the *TPS* Family in 44 Plant Species Using Conserved Domain Sequences

**Fig. 2.**
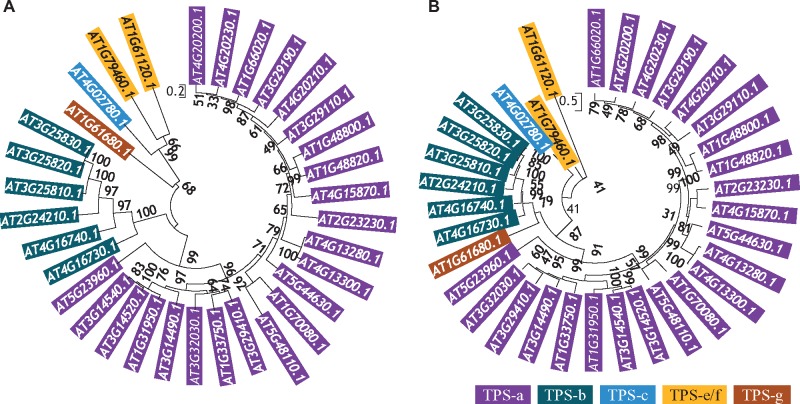
—Molecular phylogenetic analysis of *TPS*s in Arabidopsis by Maximum Likelihood method. The trees were constructed by the MEGA6 program according to the description in Materials and Methods. (*A*) and (*B*) showed the phylogenetic tree based on PF03936 and PF01397 domain sequences, respectively. The subfamilies highlighted by colors purple, benzo, blue, orange, and brown, indicate *TPS-a*, *-b*, *-c*, *-e/f*, and *-g*, respectively.

A total of seven subfamilies of *TPS*s have been identified (Chen et al. 2011). They were classified by phylogenetic analysis using full-length amino acid sequences. Here, we used conserved domain sequences to classify *TPS* members from 44 species. We used our approach to analyze Arabidopsis and rice genome first. Arabidopsis and rice are model plants for dicot and monocot plant species, respectively and they are members of angiosperms. We surveyed Arabidopsis *TPS*s using the domain PF01397 sequences. Similar to the results from previous study (Chen et al. 2011), a total of five subfamilies could be identified including *TPS-a*, *-b*, *-c*, *-e/f*, and *-g* (fig. 2*A*). Similar result was also observed when domain PF03936 sequences were used for phylogenetic analysis (fig. 2*B*). Our analysis showed that the Arabidopsis genome encodes 33 full-length *TPS*s including 23 *TPS-a*, 6 *TPS-b*, 1 *TPS-c*, 2 *TPS-e/f*, and 1 *TPS-g*, which is similar to the previous classification (Chen et al. 2011). We then investigated the rice *TPS*s. When PF01397 domain sequences were used for phylogenetic analysis, a total of five subfamilies were identified which were *TPS-a*, *-b*, *-c*, -*e/f*, and *-g* (top panel in supplementary fig. S1*A*, Supplementary Material online). However, only four subfamilies were found and *TPS-b* members were not identified when PF03936 domain sequences were employed for similar analysis (bottom panel in supplementary fig. S1*A*, Supplementary Material online). This is similar to the previous report where full-length TPSs were used for classification (Chen et al. 2011). In addition to these, we analyzed the species *Pic.abies*, which is a member of gymnosperms. Our data showed that *TPS*s in the species consisted of only two subfamilies based on PF01397 domain sequence analysis (top panel in supplementary fig. S1*B*, Supplementary Material online). Only one *TPS* is from the *TPS-e/f* subfamily whereas the remaining 27 members belong to the *TPS-d* subfamily. However, one more subfamily was identified when PF03936 domain sequences were used for analysis (bottom panel in supplementary fig. S1*B*, Supplementary Material online). The extra subfamily is *TPS-h* consisting of three members. By transcriptome mining using expressed sequence tags (ESTs) and full-length cDNAs, Keeling et al. (2011) identified a total of 69 unique *TPS*s but these members were from several *Picea* species. In the *Picea glauca* genome, 83 unique *TPS*s were identified; however, they were unique gene models but not gene loci (Warren et al. 2015). Several *Picea TPS*s have been functionally characterized and they were all from the *TPS-d* subfamily (Fäldt et al. 2003; Martin et al. 2004; Byun-McKay et al. 2006). In the lycophyte species *S. moellendorffii*, no *TPS-a*, *-b*, *-d*, and *-g* subfamilies were detected. The species mainly encodes *TPS-h* (8) followed by *TPS-c* (3) and *TPS-e/f* (2) based on the PF01397 domain sequences (top panel in supplementary fig. S1*C*, Supplementary Material online). A similar result was obtained when the PF03936 domain sequences were used for the phylogenetic analysis (bottom panel in supplementary fig. S1*C*, Supplementary Material online). In the moss species *P.patens*, only three *TPS-c* subfamily members were identified using the PF01397 domain sequences (top panel in supplementary fig. S1*D*, Supplementary Material online). However, these three members were classified into the *TPS-e/f* subfamily when using the PF03936 domain sequences (bottom panel in supplementary fig. S1*D*, Supplementary Material online).

Our classification showed that most angiosperms encode *TPS-a*, *-b*, *-c*, *-e/f*, and *-g* members; gymnosperms encode mainly *TPS-d* whereas *TPS-h* was found in the species *S. moellendorffii* (supplementary fig. S1*B*, Supplementary Material online), which is similar to previous study (Chen et al. 2011). For most dicots and monocots, the *TPS-a* subfamily is the largest group of the *TPS* family. However, our data also showed that some species have lost the *TPS-a* subfamily during long evolutionary period, for example, *Carica papaya* and *Citrus clementina* (fig. 1*C*). The loss of other subfamilies such as *TPS-b*, *-c*, and *-g* was also observed in some species but these usually occurred in their evolutionally closest species (fig. 1*C*). Interestingly, except for species from moss (*Sphagnum fallax* and *P.patens*), liverwort (*Marchantia polymorpha*), and algae (no *TPS*), all other species encoded the TPS-e/f subfamilies (fig. 1*C*). On the other hand, we found that the *TPS-d* subfamily is not gymnosperm-specific, other species including *Ananas comosus* and *M.polymorpha* also encode this subfamily member based on our phylogenetic analysis. Previously the *TPS-h* subfamily was only detected in *S. moellendorffii* (Chen et al. 2011). In this study, two *TPS-h* members were also found in species *M.polymorpha* (fig. 1*C*).

### Larger Size of the TPS Family Evolved from the Most Recent Common Ancestor in Dicots than in Monocots

**Fig. 3.**
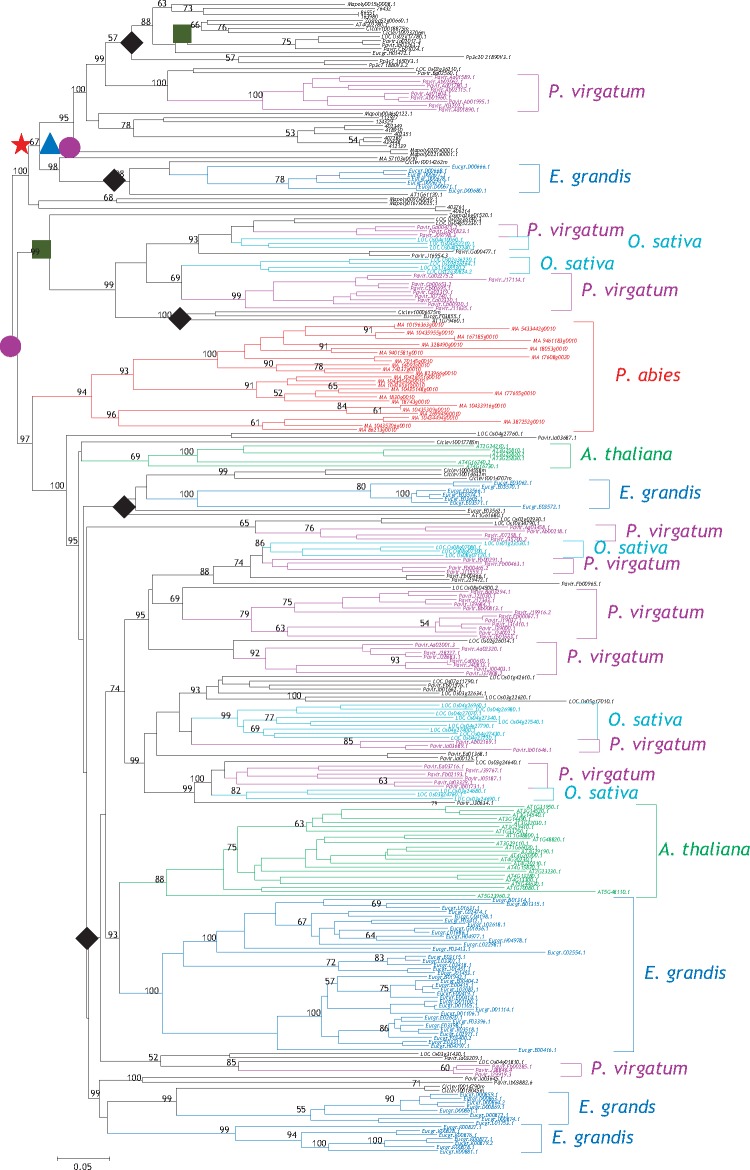
—Expansion and evolutionary history of the *TPS* family. The phylogenetic tree was constructed using the *TPS* members from ten species including liverwort (*Marchantia polymorpha*), one moss (bryophyte, *Physcomitrella patens*), one lycophyte (*Selaginella moellendorffii*), one gymnosperm (*Picea abies*), three monocots (*Oryza sativa*, *Panicum virgatum*, and *Zostera marina*), and three dicots (*Arabidopsis thaliana*, *Citrus clementina*, and *Eucalyptus grandis*). PF01397 domain amino acid sequences were employed for the tree construction using the bootstrap method with a heuristic search in the MEGA6 program. The Bayesian analyses showed a similar result. Ancestral units were defined according to Shiu et al. (2004). The red star represents the MRCA among all ten organisms and the blue triangle indicates the MRCA among moss, gymnosperm, and angiosperm. The pink circles show the MRCA units of gymnosperm and angiosperm. Blue squares and black diamond symbols represent the MRCA units in monocots and dicots, respectively. The enlarged phylogenetic tree is shown in supplementary figure S2, Supplementary Material online.

As no *TPS* members were detected in the six green algae species studied, it suggests that the family might not be essential for survival in some plants. Due to the difficulty in phylogenetic analysis using all members from 44 species, for evaluating the expansion history, we selected ten species for such an analysis (fig. 3). These species included one liverwort (*M. polymorpha*), one moss (bryophyte, *P.patens*), one lycophyte (*S. moellendorffii*), one gymnosperm (*Pic.abies*), three monocots (*Oryzasativa*, *Pan.virgatum*, and *Z. marina*), and three dicots (*Arabidopsisthaliana*, *C. clementina*, and *E. grandis*). To determine the degrees of expansion among species during long evolutionary history, we broke down the phylogeny into ancestral units according to the method described by Shiu et al. (2004). We only analyzed the expansion of full-length TPSs and lost genes and pseudogenes were excluded. Thus, the Most Recent Common Ancestor (MRCA) members may be underestimated. The analysis showed that the MRCA of liverwort, moss, gymnosperm, and angiosperm might have contained only one *TPS* (red star). No expansion occurred during the evolution to the MRCA of moss, gymnosperm, and angiosperm (blue triangle). After the origin of seed plants, one more *TPS* was found expanded in the MRCA of gymnosperm and angiosperm (filled pink circle). After this period, no expansion seems to have occurred in the MRCA of monocot plants (green square). However, three more *TPS*s expanded from the MRCA of dicot plants (black diamond). Thus, much larger size of the *TPS* families has evolved from the MRCA in dicots than in monocots. These data demonstrate that both monocot and dicot plants exhibit differences in *TPS* family expansion history. Further, many members from one species tend to cluster together, exhibiting lineage-specific expansion. A large scale of family expansion might occur during species divergence. In figure 3, species-specific expansions have been labeled with different colors. For example, in Arabidopsis, a total of two clusters were found to exhibit species-specific expansion as shown in green fonts, which formed the subfamilies *TPS-a* and *TPS-b*. In *E. grandis*, the largest cluster consists of 38 *TPS* members and all of them belong to the subfamily *TPS-a*. Thus, subfamilies were mainly expanded through species-specific expansion.

### Significant Contribution of Both Tandem and Segmental Duplication to the Family Expansion

**Fig. 4.**
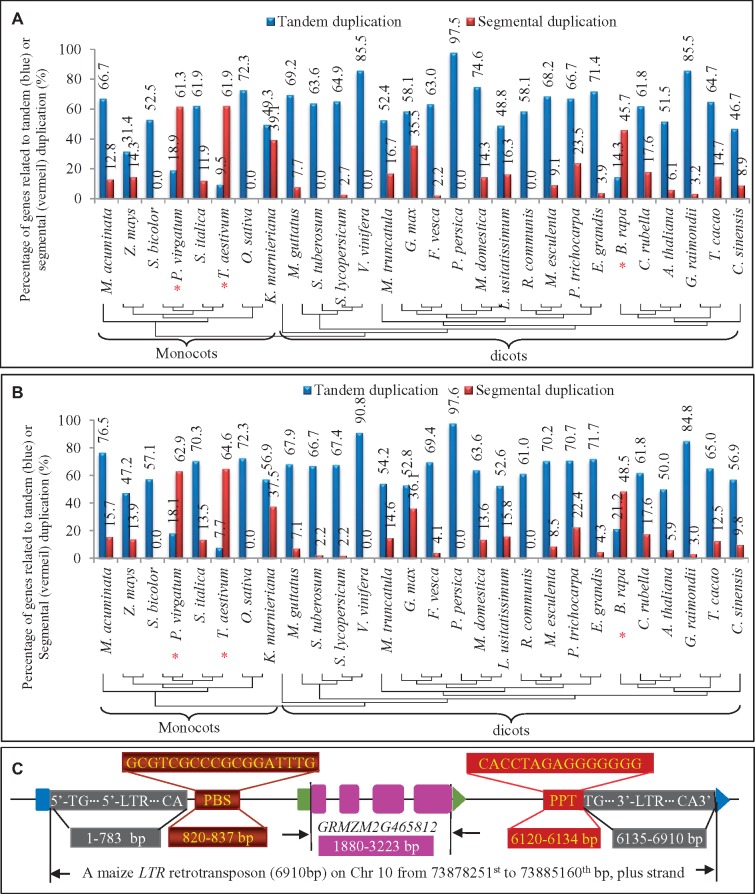
—Expansion mechanisms of the *TPS* family. (*A*) and (*B*) The effects of tandem and segmental duplications on the expansion of both domains PF01397 and PF03936, respectively. Only the species, which encode 30 or more PF01397 and PF03936 domains, were selected for expansion analysis. Blue and red curves in (*A*) and (*B*) indicate the tandem and segmental duplication, respectively. The red stars “*” indicate the species with higher ratios of segmental duplication when compared with tandem duplication. (*C*) LTR-retrotransposon mediated gene expansion. A maize *TPS GRMZM2G465812* was located within a 6,910-bp long LTR-retrotransposon. Both 5′- and 3′-LTR were highlighted with black boxes. PBS and PPT were highlighted by brown and orange boxes. Exons in the *TPS* gene were indicated by pink boxes. Blue and green boxes showed the 5′ of the retrotransposon and the *TPS* gene, respectively. Blue and green arrows indicated the end (3′) of the retranstransposon and the *TPS* gene, respectively.

We observed species-specific expansion of some *TPS* subfamilies and found large expansion mainly in dicots and monocots (fig. 3). To explore the possible mechanisms of these subfamily expansions, we first analyzed the contributions of both tandem and segmental duplications to the expansion. As the expansion rates were low in most of the non-angiosperms, we identified tandemly or segmentally duplicated genes in each species of monocot and dicot plants. We examined a total of 9 monocot and 24 dicot species whose genomes encoded at least 25 full-length *TPS*s. Since in some cases, both PF01397 and PF03936 domains were duplicated independently, we surveyed these two domains separately. For most species, tandem duplication of the PF01397 domain regions played an important role in the subfamily expansion and more than half of *TPS*s were related to tandem duplication (fig. 4*A*). However, for some of species, segmental duplication played more important role in the family expansion. For example, in the species *Brassicarapa* from dicot, *Pan.virgatum* and *T. aestivum* from monocots, segmentally duplicated *TPS*s are more than tandemly duplicated *TPS*s (red stars in fig. 4*A*). Similar situation was also observed for another domain PF03936 (fig. 4*B*). Although the tandom or segmental duplication rates for both domains may vary, the two duplication modes can be regarded as the main mechanisms for family expansion. In majority of species, tandem duplication was the major driver for family expansion. However, for few species, segmental duplication played the main role for the expansion. In some species, both tandem and segmental duplication contributed toward family expansion (fig. 4*A* and *B*). Thus, either tandem or segmental duplication or both of them can be regarded as the main mechanisms for the *TPS* family expansion.

In addition to tandem and segmental duplications, other mechanisms can also contribute to family expansion. We examined the role of various mobile elements including long terminal repeat (LTR)-retrotransposons, retrogenes, mutator-like transposable element (MULE), *hobo/Ac/Tam3* (*hAT*), *CACTA*, and *Helitron* families toward this. Our data showed that few family members were expanded by these mobile elements indicating a limited contribution of these elements to family expansion. Compared with other species, less contribution of both tandem and segmental duplication to the maize *TPS* expansion was observed (fig. 4*A* and *B*). In the species, 45.7% (PF01397) and 61.1% (PF03936) of *TPS*s were related to either tandem or segmental duplications. We examined the distribution of LTR-retrotransposons of 30 sequence fragments flanking the full-length maize *TPS*s. We found that nine *TPS*s (30%) were fully or partially located in a LTR-retrotransposon. One of the examples is shown in figure 4*C*. A 6,910 bp of typical LTR-retrotransposon is located on chromosome 10 from 73878251st to 73885160th bp (fig. 4*C*). The mobile element starts with a 783 bp of 5′-LTR and ends with a 776 bp of 3′-LTR. The *TPS* gene *GRMZM2G465812* is located between primer-binding site (PBS) and polypurine tract (PPT). Besides LTR-retrotransposons, we also surveyed the contributions of other mobile elements to the maize *TPS* family expansion. The data showed that except for one *CACTA* element, no other mobile elements were related to the family expansion.

### Plant *TPS*s Might Have Evolved from Isoprenyl Diphosphate Synthase Genes

**Fig. 5.**
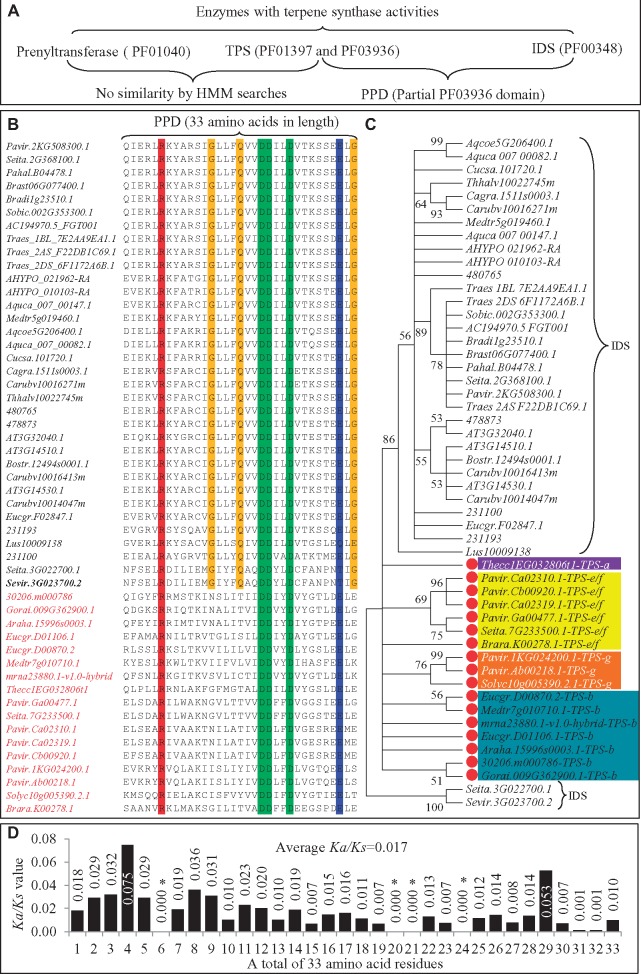
—Enzymes with terpene synthase activities and their relationships with TPSs. (*A*) Reported enzymes with terpene synthase activities. Three enzymes were listed with terpene synthase activities including prenyltransferase, TPS, and IDS. No sequence similarity was detected between prenyltransferase and TPS. However, PPD motif was detected to share homology between TPS and IDS. (*B*) Amino acid sequence alignment of PPD motif among sequences from both IDSs (locus names were labeled with black fonts) and TPSs (red fonts). Amino acid residues were indicated by capital letters in the right column. Letters highlighted by red, green, and blue colors shared 100% homology among sequences from both TPSs and IDSs. Amino acid residues highlighted by yellow color share high homology among IDSs but low homology among TPSs. (*C*) Phylogenetic analysis of amino acid sequences from PPD motifs. Sequences from IDSs were indicated by right braces and the remaining sequences were from TPSs. The subfamilies TPS-a, -b, -e/f, and -g were highlighted by purple, benzo, yellow, and orange colors. (*D*) The *Ka*/*Ks* analysis of 33 PPD amino acid residues from 34 IDS and 17 TPSs by the *SLR* program as described in Materials and Methods.

Both monoterpenes and diterpenes are synthesized from cis-prenyl diphosphates, which are substrates synthesized by cis-prenyl transferase genes. Report showed that prenyltransferase with Pfam ID PF01040 also exhibited TPS activity in animals (Gilg et al. 2009). However, no similarity in amino acid sequences between prenyltransferase and TPS was detected in our analysis (fig. 5*A*). In case of plants polyprenyl diphosphate synthase (either as isoprenyl diphosphate synthase, IDS) can show TPS activity (Green et al. 2007). Recently it was reported that in animals, a new TPS family has evolved from IDSs (Beran et al. 2016). Protein domain analysis showed that these IDSs contained the Pfam domain PF00348. We identified a total of 1,054 IDSs among 50 species in this study. Among these proteins, 46 of them (4.36%) were detected with partial PF03936 domain (PPD) when the e-value cutoff was increase to 100 instead of the default value 10. These data suggest the sequence similarity in the PPD region between TPS and IDS (fig. 5*A*). Some of these 46 PPDs are redundant and 34 of them are unique. We constructed a HMM profile using these 34 PPD sequences and carried out a HMM search against all 1,054 IDSs. We found that 948 out of 1,054 (89.9%) IDS proteins contained PPDs. Further, we used the PPD HMM profile to search against all 2,562 partial or full-length TPSs from the 50 species and found 793 (24.7%) proteins with PPDs. A total of 22,375 bacterial IDSs and 103 bacterial TPSs have been deposited in the Interpro database (June 13, 2016, http://www.ebi.ac.uk/interpro/entry/IPR000092/taxonomy; last accessed July 16, 2019). We used the PPD HMM profile to search against these bacterial IDS and TPS proteins. We detected 20,113 PPDs in 22,375 IDSs (89.9%) but none were found in TPSs.

To further analyze the sequence similarity between PPDs from IDSs and from TPSs, the 34 unique PPDs (33 amino acids in length) from IDSs were then submitted to BLAST searches against all 2,562 TPSs with e-value <0.01. Only 18 TPSs were obtained from these searches with PPD homolog. These PPDs from 18 TPSs and 34 IDSs were then used for alignment analysis. A total of five amino acid sites are very conserved (100% homolog) among these 52 analyzed sequences and they are highlighted by red, green, and blue colors (fig. 5*B*). Besides the five amino acid sites, three additional sites (G, Q, and G) were observed, which were conserved only in the 34 IDSs but not in TPSs and are highlighted by yellow color (fig. 5*C*). The phylogenetic analysis using 52 PPD sequences showed that all 34 unique IDSs were grouped together, separating from the group consisting of 18 TPSs (fig. 5*C*). These 18 TPSs were from 4 different TPS subfamilies including 1 TPS-a, 7 TPS-b, 6 TPS-e/f, and 3 TPS-g. In fact, the motif “DDXXD” was detected in many subfamilies but not in TPS-c (Chen et al. 2011). Our data showed that this motif is also present in many IDS proteins.

As PPDs from both TPS and IDS fell into different groups, we studied the selection force on these 33 amino acids sites among different species by using the sitewise likelihood-ratio (SLR) program (Massingham and Goldman 2005). Both aligned coding sequences and the phylogenetic tree were submitted to the SLR analysis (fig. 5*D*). A total of four amino acid sites (as indicated by “*” in fig. 5*D*) showed nonsynonymous substitution with *Ka*/*Ks* = 0. The most variable site was the fourth amino acid with *Ka*/*Ks *=* *0.075. The average *Ka*/*Ks* is 0.017. The data reveal strong purifying selection of PPD sites even among different orders of species. IDSs are widely present in human/animals, plants, fungi, bacteria, and even in viruses while TPSs are mainly in plants, fungi, and bacteria. Some IDSs do exhibit TPS activities and we found that they have the common PPD motif. This suggests that *TPS* might have evolved from *IDSs*.

### Domain Loss Occurred Frequently in Some Species

**Fig. 6.**
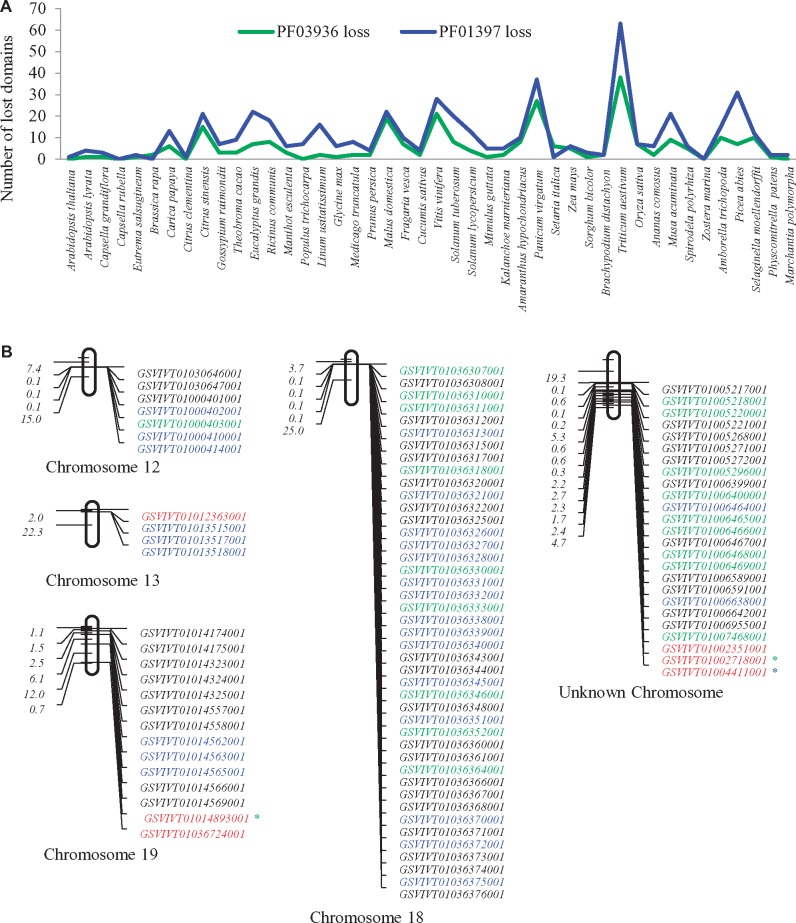
—Detection of domain loss and its possible mechanisms. (*A*) General profiles of both PF01397 and PF03936 domain loss in 44 plant species. (*B*) Chromosomal distribution of *TPS*s and detection of tandemly/segmentally duplicated genes in *Vitis vinifera*. Genes marked with red fonts indicated nontandemly duplicated genes and the remaining genes were all related to tandem duplication. Genes with PF01397 or PF03936 domain loss were labeled with blue or green fonts, respectively. The first value in each chromosome represents the physical position (Mb) of mapped genes. The second value shows the physical distance to the first gene. The rest may be calculated by analogy. Green star “*” indicates that the corresponding gene suffered from PF01397 loss but was not tandemly duplicated gene. The blue star “*” indicates the genes with PF03936 loss that occurred in nontandem duplicated region.

Detailed examination of all identified TPSs and their domain composition showed that many TPSs contained only either PF01397 or PF03936 domain. For example, in the species *Citrus sinensis*, up to 45 PF01397 and 51 PF03936 domain encoding *TPS*s genes were identified; however, only 30 out of them were full-length *TPS*s with two domain structures (fig. 1). Generally, a typical TPS functions in plants by two domains. Thus, 15 TPSs lost the domain PF03936 and 21 genes lost PF01397 domain. We then further surveyed the domain loss in all 44 species (fig. 6*A*). Generally, PF01397 domain loss occurred more frequently than the PF03936 domain (fig. 6*A*). Our data showed that domain loss occurred in majority of species and it corresponds to family sizes. The larger the family size is the more domains lost. For instance, both *Triticum aestivum* and *Pan.virgatum* contain large size *TPS* families and they also have more partial *TPS*s with only either PF01397 or PF03936 (fig. 6*A*). In wheat species, 38 TPSs lost the domain PF01397 and 63 TPSs lost the other. Similarly, in switch grass, 27 and 37 of TPSs lost PF01397 and PF03936, respectively. These data show domain loss as a universal event during family evolution of TPSs.

To examine the widespread event of domain loss, we selected a relatively large *TPS* family from *V.vinifera* for further analysis. In this species, we identified at least 78 PF01397 and 76 PF03936 domain encoding genes and 57 of them contain two domains. Thus, 21 TPSs lost PF03936 and 19 TPSs lost PF01397 domain. Except for *GSVIVT01033458001*, all partial TPSs were located on chromosomes 12, 13, 18, and 19, or on unknown chromosome (fig. 6*B*). In these chromosomes, six *TPS*s were not related to tandem duplication (labeled with red fonts in fig. 6*B*) but the remaining *TPS*s were subjected to tandem duplication. Thus, most of partial *TPS*s arose from tandem duplication and domain loss might have occurred during this event by duplicating a fragment encoding single domain. However, in some species, tandem duplication was not the major force to contribute to family expansion (fig. 4*A* and *B*). Hence, we investigated the contribution of segmental duplication to domain loss. We selected the wheat species for this analysis as segmental duplication has played important role in family expansion. Our data showed that in this species, up to 87% of partial TPSs were located on segmental duplication blocks. This indicates that domain loss can also occur during segmental duplication. Domain loss might be over- or underestimated due to the missing during the identification of TPS domains using our methods or inaccurate prediction of TPS domains.

### Expression Divergence of the *TPS* Families in Different Species

**Fig. 7.**
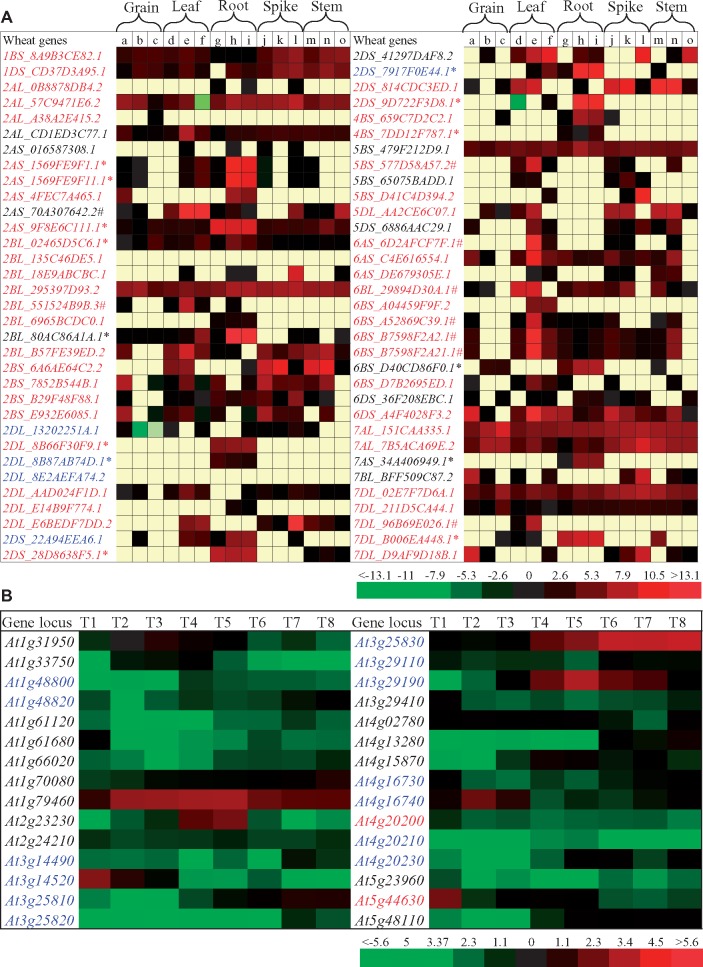
—Expression profiles of *TPS*s. (*A*) Expression patterns of the wheat *TPS*s. The expression data were based on the RNA-Seq data from the European Nucleotide Archive database with accession number ERP004714. The yellow color cells indicate the unit of fragments per kb of exon per million mapped reads (FPKM) was zero with no expression in this tissue. The transcript abundance in the expressed tissues in the heat map was estimated by the value from Log_2_(FPKM). The symbols “*” and “#” indicated root- and leaf-preferred genes, respectively. (a) Grains at kernel watery ripe stage; (b) Grains at medium milk stage; (c) Grains at soft dough stage; (d) Leaves at first leaf through coleoptile stage; (e) Leaves at main shoot and three tillers stage; (f) Leaves at kernel watery ripe stage; (g) Roots at first leaf through coleoptile stage; (h) Roots at three leaves unfolded stage; (i) Roots at flag leaf ligule and ollar visible stage; (j) Spikes at second detectable node stage; (k) Spikes at flag leaf ligule and ollar visible stage; (l) Spikes at 1/2 of flowering complete stage; (m) Stems at pseudostem erection stage; (n) Stems at second detectable node stage; and (o) Stem at 1/2 of flowering complete stage. (*B*) Heap map showing the expression profiling of Arabidopsis *TPS*s in eight different tissues. The microarray data were downloaded as described in Materials and Methods. The processed expression values were calculated from three biological repeats and were then converted into log2 scale, which were used for the heat map. T1, siliques at Stage 3; T2, siliques at Stage 4; T3, siliques Stage 5; T4, Seeds at Stage 6; T5, Seeds at Stage 7; T6, Seeds at Stage 8; T7, Seeds at Stage 9; T8, Seeds at Stage 10. The red and blue locus names in (*A*) and (*B*) indicated segmentally and tandemly duplicated genes, respectively.

We were interested to know how these genes survived after the large scale of expansion by duplication. Our data showed that the *TPS* families mainly expanded by either segmental or tandem duplication. We selected two species, wheat and Arabidopsis for expression analysis studies. In wheat *T.aestivum*, segmental duplication was found to be the main force for family expansion (fig. 4). RNA-Seq expression data from 15 different tissues were downloaded as described in Materials and Methods. These tissues were from different stages of grains, leaves, roots, spikes, and stems (fig. 7*A*). Among the total of 67 full-length *TPS*s identified in this species, no RNA sequence information was available for the member *Traes_3B_4BFFDC15C* (The prefix “*Traes_*” was omitted thereafter for convenience). Expression data for the remaining 66 TPSs is shown in figure 7*A*. No significant expression signal in any of 15 tissues was detected for two *TPS*s including *2BL_135C46DE5.1* and *2DL_8E2AEFA74.2* (fig. 7*A*). Interestingly, we detected 14 root-preferred and 7 leaf-preferred genes as indicated by “*” and “#” in figure 7*A*, respectively. These genes showed at least two times higher expression in either roots or leaves when compared with the remaining tissues, suggesting tissue-specific roles for these genes. We also surveyed the expression divergence among segmentally or tandemly duplicated genes. We found by statistical analysis (*P* < 0.01) that all duplicated genes showed expression divergence either in expression abundance in a specific tissue or in tissue specificity. For example, both *2DL_8B87AB74D.1* and *2DL_8E2AEFA74.2* were tandemly duplicated; the former showed root-preferred expression and the latter showed no expression in all tested tissues. Another example is the segmentally duplicated genes *1BS_8A9B3CE82.1* and *7BL_BFF509C87.2*, they showed totally different expression patterns.

In *A.thaliana*, tandem duplication is the main force for the family expansion. In this species, a total of eight tissues were selected for expression analysis using microarray data as described in the methods. These tissues were sampled from eight different developmental stages (Stage 3 to Stage 10) (Boyes et al. 2001). Similarly, we detected several developmental stage-preferred genes. For example, both *At3g14520* and *At5g44630* showed highest expression level at the stage 3 while the gene *At4g16740* showed the highest expression in Stage 4 (fig. 7*B*). Both *At4g20200* and *At5g44630* were segmentally duplicated and they showed significant difference in their expression patterns among the eight tissues (fig. 7*B*). Tandemly duplicated genes also showed statistical difference in their expression abundance or showed expression divergence in tissue specificity (genes with blue fonts in fig. 7*B*). Thus, the expression profiles in both wheat and Arabidopsis further confirmed that expression divergence occurred after either tandem or segmental duplication.

### Analysis of the *TPS* Families in Mint and Basil by Transcriptomics

Aromatic plants mint and basil produce essential oils rich in terpenes in organs called PGT (Mimica-Dukic and Bozin 2008; Chenni et al. 2016). Although the whole genome sequence is available for the species *O.tenuiflorum* (Upadhyay et al. 2015) and *Ocimum sanctum* (Kumar et al. 2018), genome sequencing data are not available for many aromatic plant species and therefore, some of TPSs were identified by transcriptomic analysis via RNA-Seq (Tsaballa et al. 2015; Hu et al. 2016; Meena et al. 2016). RNA-Seq was carried out using total RNA samples isolated from four different tissues namely PGTs, leaves stripped of PGTs (leaf-PGTs), leaves, and roots. In this study, we carried out a HMM search against the transcriptomic data set and identified 34 *TPS*s of which 17 were full-length *TPS*s, 11 partial *TPS*s encoding only PF01397 domain and 6 partial *TPS*s with only PF03936 domain (supplementary fig. S3*A*, Supplementary Material online). Their unigene names and corresponding deduced amino acid sequences are listed in the supplementary table S2, Supplementary Material online. We also generated RNA-Seq data from four different basil (*O.basilicum*) tissues similar to mint. Based on the transcriptomic data set, we identified 25 full-length *TPS*s, 9 partial *TPS*s encoding PF01397 domain only and 8 partial *TPS*s with PF03936 domain only (supplementary fig. S3*B*, Supplementary Material online). Their unigene names and corresponding deduced amino acid sequences are listed in the supplementary table S3, Supplementary Material online. In addition, since the draft whole genome sequence of the basil species *O.tenuiflorum* has been published (Upadhyay et al. 2015), we further identified the TPSs based on the whole genome data. A total of 57 and 69 putative *TPS*s encoding PF01397 and PF03936 domains, respectively were found. Among them, 42 *TPS*s encoded both the domains (supplementary fig. S3*C*, Supplementary Material online). Their locus names and corresponding amino acid sequences are given in the supplementary table S4, Supplementary Material online.

Among the 17 full-length mint *TPS*s, three of them had premature stop codons or frameshifts and they might have evolved into pseudogenes. These putative pseudogenes were excluded for phylogenetic analysis as they might affect the sequence alignment. For the mint *TPS* family, the phylogenetic trees from both domains PF01397 and PF03936 are similar (supplementary fig. S3*D* and *E*, Supplementary Material online). The family consists of four different subfamilies including three *TPS*s*-a*, five *TPS*s*-b*, four *TPS*s-*e/f*, and two *TPS*s*-g*. For basil, 42 TPSs were used for the phylogenetic analysis. The analysis showed that the family could also be classified into four subfamilies including *TPS-a*, *-b*, -*e/f*, and *-g* (supplementary fig. S3*F* and *G*, Supplementary Material online). However, both domains PF01397 and PF03936 showed differences in the subfamily classification when they were used for phylogenetic analysis. The gene *Ote100217510041* (indicated by “*”) and *Ote100185690021* or *Ote100079410021* (indicated by “#”) clustered into *TPS-a*, when the PF01397 domain sequences were used for phylogenetic analysis (supplementary fig. S3*F*, Supplementary Material online). However, the gene labeled by “*” was grouped into *TPS-g* and the remaining two genes marked by “#” clustered into *TPS-b*, respectively, when the PF03936 domain sequences were used for the phylogenetic analysis (supplementary fig. S3*G*, Supplementary Material online). These data suggest inconsistent evolution between these two domains in the basil *TPS* family. This inconsistence might also be due to incorrect assembly.

We surveyed the expression profile of these *TPS*s including partial *TPS*s. In mint, a total of 29 *TPS*s showed expression evidence in at least one of three tissues and some showed preferential expression in certain tissues (supplementary fig. S4*A*, Supplementary Material online). We detected three root-preferred and three leaf-PGT-preferred *TPS*s (indicated by “*,” supplementary fig. S4*A*, Supplementary Material online). Interestingly, majority of *TPS*s showed enhanced expression in PGT (supplementary fig. S4*A*, Supplementary Material online). PGT are the sites of terpene biosynthesis in this plant and these *TPS*s provide candidate genes for further improving terpene synthesis. Among 12 (41.4%) PGT-preferred TPSs, 8 *TPS*s encoded both PF01397 and PF03936 domains. Four PGT-preferred *TPS*s encoded only one domain and three of them contained only the PF01397 domain including *comp26858_c1*, *comp35437_c0*, and *comp43257_c0* and the remaining one *comp34823_c3* encoded the PF03936 domain. However, this is only based on RNA-Seq assembling result and further experiments such as RACE (Frohman et al. 1988) should be carried out to confirm the length of these *TPS*s.

For the basil *TPS*s, we also carried out the expression abundance analysis among the three different tissues (supplementary fig. S4*B*, Supplementary Material online). Different from the expression data of mint (supplementary fig. S4*A*, Supplementary Material online), all 42 partial or full-length *TPS*s showed detectable expression signal in either one or the three tested tissues. Three *TPS*s (unigenes *comp79982_c0*, *comp72455_c0*, and *comp105854_c0*) showed expression signal only in roots (supplementary fig. S4*B*, Supplementary Material online). Interestingly, more *TPS*s were detected with PGT-preferred expression pattern. Totally, we identified eight root-preferred and three leaf-PGT-preferred YPSs (supplementary fig. S4*B*, Supplementary Material online). Most of the *TPS*s (about one-third, 14 *TPS*s) showed PGT-preferred expression pattern. These data suggest the tissue-specific functions of some *TPS*s toward producing specialized metabolites in specific organs such as PGTs for plants ecological benefit.

### The Origin of the *TPS* Family and Putative Horizontal Gene Transfer

Evidence shows that *TPS*s in plants, fungi, and bacteria shared a common evolutionary origin since they have similar reaction mechanism, conserved domain organization, intron, and exon structures (Trapp and Croteau 2001; Morrone et al. 2009; Fischer et al. 2015; Singh and Sharma 2015). These studies also showed molecular evidence of horizontal gene transfer (HGT) events. Generally, *di-TPS*s were horizontally transferred from soil bacteria to plants. Recent evidence also showed the HGT event of *TPS* from a plant to a fungus (Fischer et al. 2015). Interestingly, in this study, we found a clue of a possible HGT event of *TPS* from a plant to a bacterium. In the Interpro database (June 16, 2016, http://www.ebi.ac.uk/interpro/; last accessed July 16, 2019), a total of 103 protein sequences has been deposited, which contain the PF03936 domain structure. We downloaded these protein sequences and then submitted to BLASTP search against all TPS proteins that were identified from the 50 species. We detected two bacterium proteins showed high-sequence similarity to plant *TPS*s. One of them is with protein ID A0A0C3RJ57 in the database (175aa), which showed 100% homolog (1-175aa) to a cotton protein with locus name *Gorai.009G428400.1*. Another one is A0A0C3RA45 (177aa), which showed 97.2% of identity (1-177aa) to the cotton protein sequence with locus name *Gorai.009G428200.1*. In the cotton genome, these two loci were tandem duplicated. Both A0A0C3RJ57 and A0A0C3RA45 are from the bacterium *Nitrosospira sp NpAV*. Its genome is fully sequenced and encodes 3,242 annotated genes (http://www.ncbi.nlm.nih.gov/genome/17307; last accessed July 16, 2019). The genome-wide survey identified only two members of TPSs (A0A0C3RJ57 and A0A0C3RA45) in the bacterium. Three other genomes from *Nitrosospira* have also been sequenced and they are *Nitrosospira multiformis*, *Nitrosospira briensis*, and *Nitrosospira lacus*. All these three genomes do not encode any *TPS* based on our HMM/BLASTP searches against these protein data sets. In addition, no significant homologs of both A0A0C3RJ57 and A0A0C3RA45 (identities > 50%) could be found in any other bacteria in the Interpro database. On the contrary, many homologous *TPS*s could be detected among plant species when these two members were used as query for BLASTP searches against plant genomes. Thus, our analysis provided molecular evidence that these two members of bacterium *TPS*s might be horizontally transferred from the plant species *Gossypium raimondii*. However, no experimental evidence was obtained. It is possible that the observed results might be due to sample contamination; thus, further study should be carried out to confirm the HGT event.

Our data showed ubiquitous distribution of *TPS*s among plants, fungi, and bacteria and at least one MRCA was detected among the 50 plant species. On the other hand, little is known as to why animals contain no *TPS*. Most of our knowledge on *TPS*s comes from plant studies. However, some animals have the ability to synthesize terpene-derived secondary metabolites. Evidence shows that these terpenes are synthesized by either prenyltransferase (Gilg et al. 2009) or IDSs (Beran et al. 2016). The data from the report showed the evolution of animal *TPS*s from *IDSs* (Beran et al. 2016). Our data showed that some of plant TPSs shared common PPD motif with animal IDSs (fig. 5). In some of plants, IDSs are bifunctional enzymes with both prenyltransferase and TPS activity (Yang et al. 2014). The data together with the ubiquitous distribution of IDSs in all organisms suggest that plant *TPS*s might have also evolved from *IDSs*.

## Discussion

In this study, we screened for *TPS* family members from 50 species (fig. 1). These species ranged from green algae, liverwort, bryophytes, gymnosperms to angiosperms. *TPS* family members could be detected in only 44 species. The remaining six species encoded no *TPS* and they all belonged to green algae. We also checked for the presence of TPSs in additional 12 algal genomes but no TPSs were identified. Recently, Jia et al. (2016, 2018) identified microbial TPS-like (MTPSL) genes in 158 species from either charophytes or chlorophytes and only one species from charophytes encodes *MTPSL* and they were regarded as new TPSs in nonseed plants. All these data suggest the lack of *TPS*s in most of alga species. However, *TPS*s were widely distributed in bacteria (Yamada et al. 2015). TPSs could also be found in fungi (Fischer et al. 2015). BLAST searches revealed the presence of *TPS*s in two Metazoa species *Dictyocaulus viviparous* and *Trichinella nelson*. Thus, TPSs are ubiquitous among bacteria, fungi, liverworts, bryophytes, gymnosperms, and angiosperms but are present in only few of Metazoa species. No *TPS*s were detected in algae and animals. In a broad sense, TPSs can be classified into class I and class II (Tholl 2006; Thimmappa et al. 2014). Both oxidosqualene cyclase and squalene-hopene cyclase have been regarded as class II of TPSs (Thimmappa et al. 2014). They contain no PF01397 and PF03936. Some of ent-copalyl diphosphate synthases are squalene-hopene cyclases, which also contain no PF01397 and PF03936. In addition, IDS also show TPS activity in plants and animals (Green et al. 2007; Lancaster et al. 2019). Thus, algae and animals might use other TPSs without either PF01397 or PF03936 domain for terpene biosynthesis.

Genome-wide identification of the *TPS* family members have been carried out in many species. The numbers of identified family members can vary in different studies due to 1) different genome assemble and annotation version, 2) either PF01397 or PF03936 included, 3) different search methods, and 4) different databases. For example, Martin et al. (2010) identified 69 putatively functional *TPS*s in *V.vinifera* (Martin et al. 2010); however, only 57 full-length TPSs were identified in this study (fig. 1*A*). Detailed comparison showed that additional 12 members were identified using the genomic DNA sequence data, which were not included in the protein database used by us. Similar situation was also observed for the *TPS* family in *E. grandis*, where a total of 113 partial or full-length *TPS*s were identified (Külheim et al. 2015). Kumar, Kempinski, et al. (2016) identified 13 putative *TPS*s with complete reading frames from *M. polymorpha* transcriptomes but not from annotated protein database. However, these may include isoforms. Apart from identification from single species, genome-wide identification of *TPS*-related genes from multiple genomes have also been reported. For example, 17 genomes were employed to investigate terpene diversification by Boutanaev et al. (2015). They focused on genes related to terpene metabolism with coevolution relationships; thus, not all *TPS*s were identified. Hofberger et al. (2015) identified core *TPS*s from 17 genomes. They did not investigate their domains separately. In this study, we identified *TPS*s from a total of 50 species using HMM profiling searches against annotated protein databases. Thus, some putative *TPS*s, which were not annotated in the databases due to various reasons, could not be included in this study. Additionally, we identified *TPS*s by detecting both domains PF01397 and PF03936 separately, thus, minimizing the chance of missing putative *TPS*s. Therefore, our investigation on evolutionary and expansion history of this family should provide a more comprehensive view due to the increased numbers of species and improved genome-wide identification.

We also investigated the evolutionary patterns of *TPS* family in the 44 species. This family is a midsize family as identified by Chen et al. (2011). Some species like *Z.marina* has only two members and few other species also encode very less *TPS*s (fig. 1). In contrast, some species have evolved large *TPS* family consisting of more than 100 members (fig. 1). The large scale of gene family expansion can occur in different evolutionary stages for different gene families. For the receptor-like kinase family, the large-scale expansion occurred before the diversity of Arabidopsis from rice (Shiu et al. 2004). For the *TPS* family, the large-scale expansion mainly occurred after species diversity. Species-specific expansion significantly contributed to the differentiated expansion rates among species (fig. 3). For example, high ratios of *TPS*s have expanded in *E. grandis* and *Pan.virgatum*, as a result, their *TPS* families consisted of 70 or more members (figs. 1 and 3). Species-specific expansion of other gene families has been observed in many other species. For example, a terpene synthesis-related gene family, the 3-hydroxy-3-methylglutaryl coenzyme A reductase (HMGR) also experienced species-specific expansion (Li et al. 2014). However, their expansion mechanisms might vary from species to species and for different gene families. Some gene families can be expanded by mobile elements (Hoen et al. 2006; O’Toole et al. 2008). Others might be mainly expanded by tandem or segmental duplication (Chi et al. 2011; Jiang et al. 2013). For the *TPS* gene family, different species showed difference in their expansion mechanisms. For most species, tandem duplication played most important role in the family expansion (fig. 4). However, for some species, for example, *Pan.virgatum*, *T.aestivum*, and *B.rapa*, segmental duplication is the main driver for family expansion (fig. 4). In other species, tandem and segmental duplication as well as mobile elements contributed to family expansion. Thus, *TPS*s expanded themselves through different mechanisms in different species.

In the Interpro database, only four bacterium sequences were detected with TPS N-terminal domain while 103 bacterium sequences had the TPS C-terminal domain. Thus, majority of bacterium TPSs contain only the C-terminal domain. In plants, majority of TPSs contain both domains, suggesting a substantial difference between plant and bacterium TPSs. In plants, domain loss for either PF01397 or PF03936 frequently occurred in many species (fig. 6), which was likely triggered by partial duplication. The functionality of these single domain-containing TPSs are not known. The phylogenetic trees derived from both domains showed significant difference for some species (supplementary figs. S1 and S3, Supplementary Material online). This raises the issue of selection of domain sequences for phylogenetic analysis and family classification. Currently, majority of the work is carried out by using the full-length TPSs. However, both domains showed difference in their biological functions. Thus, it will be better to use either N- or C-terminal domain sequences separately to construct phylogenetic trees for evolutionary and functional analysis. Such analysis might lead to minor difference in family classification but will provide a better understanding of the difference in their biological functions.

For some species, only a few members of *TPS*s are required. For example, the aquatic plants *Z.marina* and *Spirodela polyrhiza* encode only two and six *TPS*s, respectively. Similarly, the *TPS* family size for plants from bryophyte and liverwort is also small (fig. 1). For these species, less expansion was observed and they contained no *TPS-a* and *TPS-b*. The *TPS* expansion occurred mainly in both monocot and dicot plants. The expansion history showed that monocot MRCA contained only two members while dicot MRCA required three more *TPS*s. The large-scale of expansion occurred after species diversification and many species-specific *TPS*s have expanded (fig. 3). Most of expanded *TPS*s belong to *TPS-a* and *TPS-b* subfamilies (fig. 1). Thus, different plant species required different subfamilies of *TPS*s for their secondary metabolite needs and exhibited different expansion history. This suggests that *TPS* expansion might be related to species or ecotype diversification or environmental adaption.

We further carried out expression profiling on this gene family in four different species. The monocot wheat species showed a relative larger expansion mainly by segmental duplication (fig. 7*A*). The dicot Arabidopsis species exhibited middle level of gene expansion rate mainly by tandem duplication (fig. 7*B*). The remaining two species were aromatic plants (supplementary fig. S4, Supplementary Material online). Our expression analysis showed that most of duplicated genes were expressed in either single or multiple tissues. Duplicated genes showed significant expression divergence either in tissues or transcript intensity and no gene was expressed with the same expression abundance or tissue specificity. Thus, duplicated genes carried on with similar functions but exhibited divergent expression patterns. Generally, our expression data suggest that expression divergence significantly contributed to gene survival after gene expansion by duplication or mobile elements.

In wheat, a total of 14 root-preferred and 7 leaf-preferred *TPS*s were detected (fig. 7*A*). Some of Arabidopsis *TPS*s also showed developmental stage-preferred expression (fig. 7*B*). Previous studies have reported the tissue-specific expression of *TPS*s. For example, flower-, seed-, stem-, and root-specific genes have been identified in both Arabidopsis and *Medicago* (Parker et al. 2014). In aromatic plants like chamomile, some *TPS*s are involved in organ-specific formation of essential oils (Irmisch et al. 2012). Thus, some plant *TPS*s show tissue-specific functions. Although both mint and basil belong to same family, they showed differences in the expansion and evolution of *TPS* family (supplementary fig. S3, Supplementary Material online). These two species also exhibited difference in *TPS* expression patterns (supplementary fig. S4, Supplementary Material online). Essential oils are highly accumulated in glandular trichomes (Wang et al. 2008). Thus, identification of trichome-preferred genes would significantly contribute to the understanding of terpene synthesis. We have detected 12 and 14 PGT-preferred *TPS*s in mint and basil, respectively (supplementary fig. S4, Supplementary Material online). Thus, genetic manipulation of expression abundance in these *TPS*s would contribute to improved terpene metabolism. PGT-preferred genes provide candidates for us to genetically modify the essential oil accumulation in trichomes.

## Conclusions

In the present study, both N- and C-terminal domain sequences of seed TPSs were separately used to construct HMM profiling. They were then employed for HMM searches to genome-widely identify the *TPS* family members from 50 sequenced genomes, which were from algae, liverwort, bryophyte, lycophyte, gymnosperm, and angiosperms. We also molecularly characterized the *TPS* family from aromatic spearmint (*Mentha spicata*) and basil (*O.basilicum*) plants using our RNA-Seq data. No *TPS*s were identified in all six green algae genomes and the lack of algae *TPS*s was further confirmed by searching additional 12 algae genomes. The remaining 44 plant species encode various sizes of *TPS* family members ranging from 2 to 79 full-length *TPS*s. Phylogenetic analysis showed that these *TPS*s could be classified into seven different subfamilies including *TPS-a*, *-b*, *-c*, *-d*, *-e/f*, *-g*, and *-h*. No genome encodes all seven subfamilies of *TPS*s, suggesting the divergence of this family during long evolutionary history. Some species showed lineage-specific expansion of some subfamilies and a large-scale family expansion occurred mainly in dicot and monocot plants. Further investigation showed that both tandem and segmental duplication significantly contributed to the family expansion. Further analysis showed that expression divergence significantly contributed to gene survival after duplication or expansion. Interestingly, not only alga but also animals contain no *TPS*; however, they all encode *IDSs*. Our data provided the clue that *TPS*s might have originated from *IDSs*.

## Supplementary Material

Supplementary_Data_evz142

## Notes

**Data deposition:** This project has been deposited at the DDBJ database (http://trace.ddbj.nig.ac.jp/dra/index_e.html; last accessed July 16, 2019) under the accession numbers DRA001856. The remaining data sets supporting this study are included within the article and its Supplementary Material online.
