# Supplementary material for: A Comprehensive Survey on the Terpene Synthase Gene Family Provides New Insight into Its Evolutionary Patterns
Source: Genome Biol Evol. 2019 Jul 15;11(8):2078–98. doi: 10.1093/gbe/evz142 (PMC6681836; doi:10.1093/gbe/evz142)
Supplement: Supplementary_Data_evz142 [file supplementary_data_evz142.zip › Suppl materials legends.docx]

**Supplementary Materials**

**Supplementary table S1_Sheet1.** Genome-wide identification of genes encoding PF01397 domain in 44 plant species

**Supplementary table S1_Sheet2.** Genome-wide identification of genes encoding PF03936 domain in 44 plant species

**Supplementary table S1_Sheet3.** Genome-wide identification of genes encoding both PF01397 and PF03936 domains in 44 plant species.

**Supplementary table S2.** Unigenes encoding TPSs in the mint genome based on the RNA-Seq dataset.

**Supplementary table S3.** Unigenes encoding TPSs in the basil genome based on the RNA-Seq dataset.

**Supplementary table S4.** Genome-wide identification of genes encoding TPSs in basil based on the whole genome sequence.

**Supplementary figure S1**. The enlarged phylogenetic trees in Figure 2.

**Supplementary figure S2.** The enlarged phylogenetic tree of Figure 3.

**Supplementary figure S3.** Identification and characterization of the *TPS* family in mint and basil.

**Supplementary figure S4.** Expression profiling of TPSs in mint and bail based on RNA-Seq analysis.
