# Supplementary material for: A Comprehensive Survey on the Terpene Synthase Gene Family Provides New Insight into Its Evolutionary Patterns
Source: Genome Biol Evol. 2019 Jul 15;11(8):2078–98. doi: 10.1093/gbe/evz142 (PMC6681836; doi:10.1093/gbe/evz142)
Supplement: Supplementary_Data_evz142 [file supplementary_data_evz142.zip › Supplementary Figure S1.pdf]

Supplementary Figure S1

A

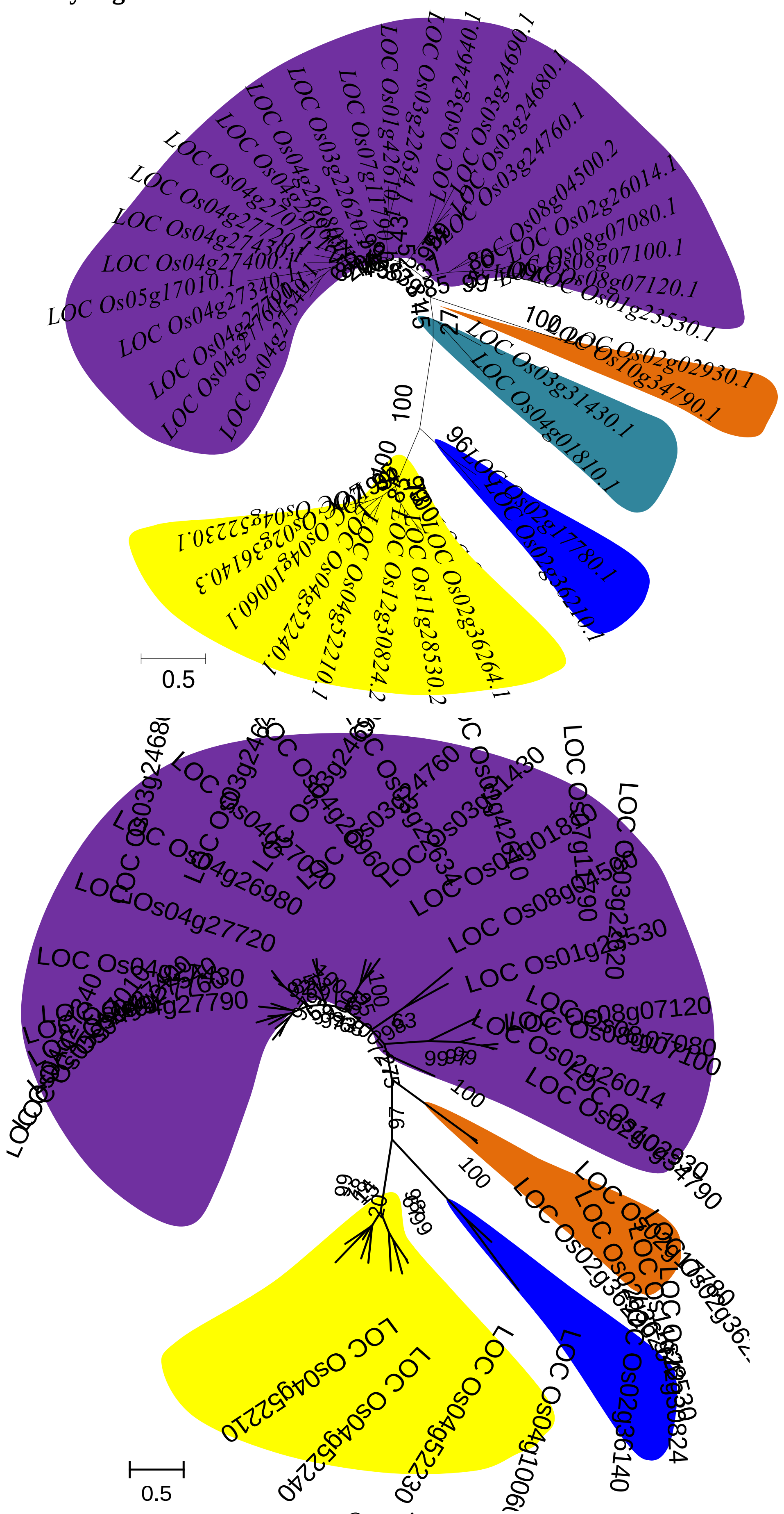

O. sativa

Supplementary Figure S1

B

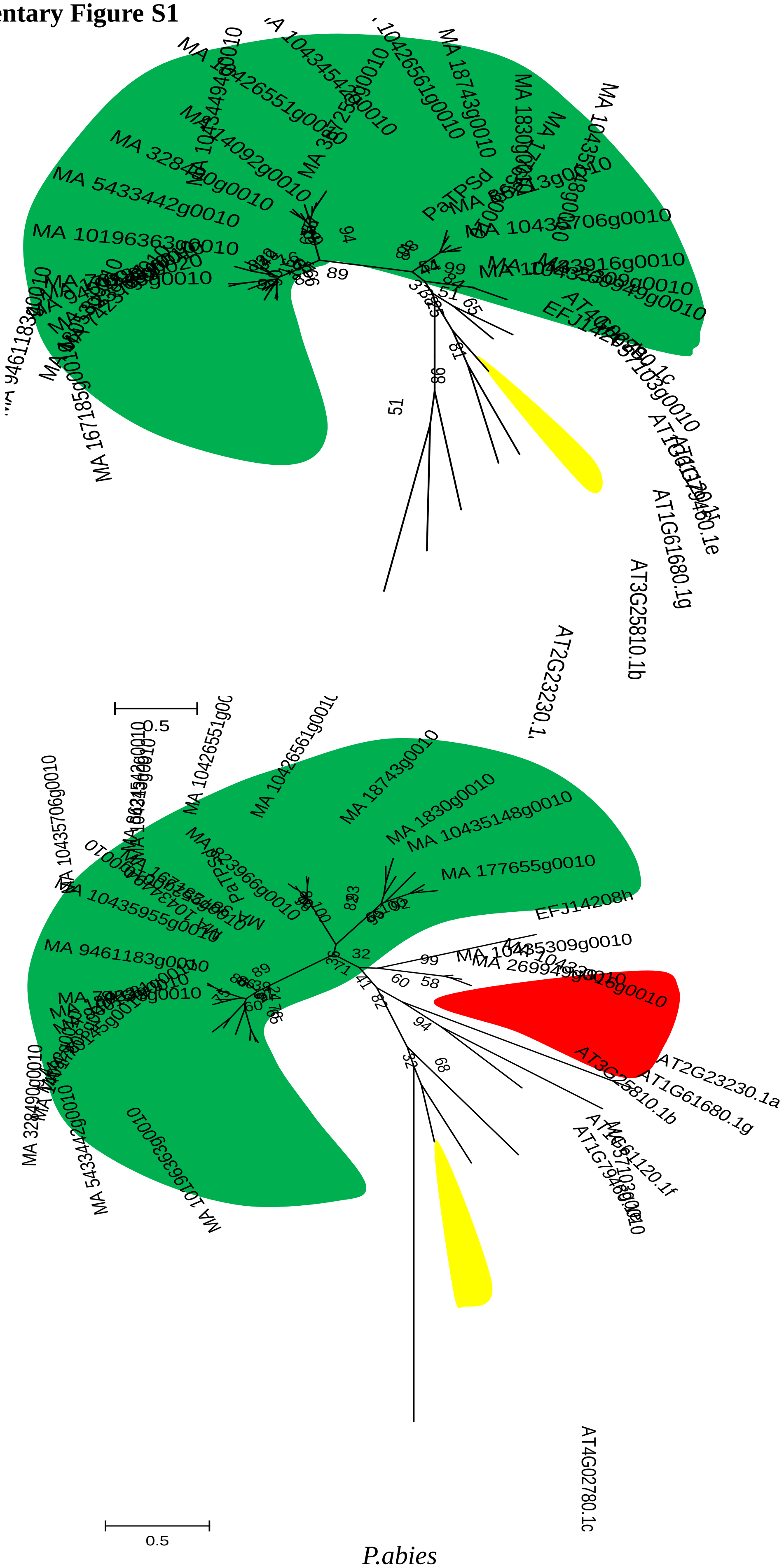

P.abies

Supplementary Figure S1

C

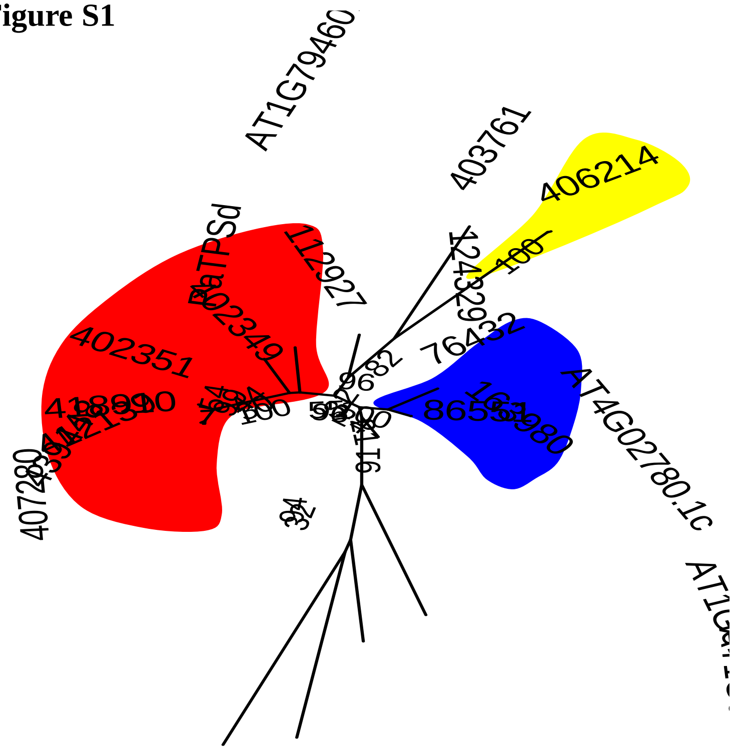

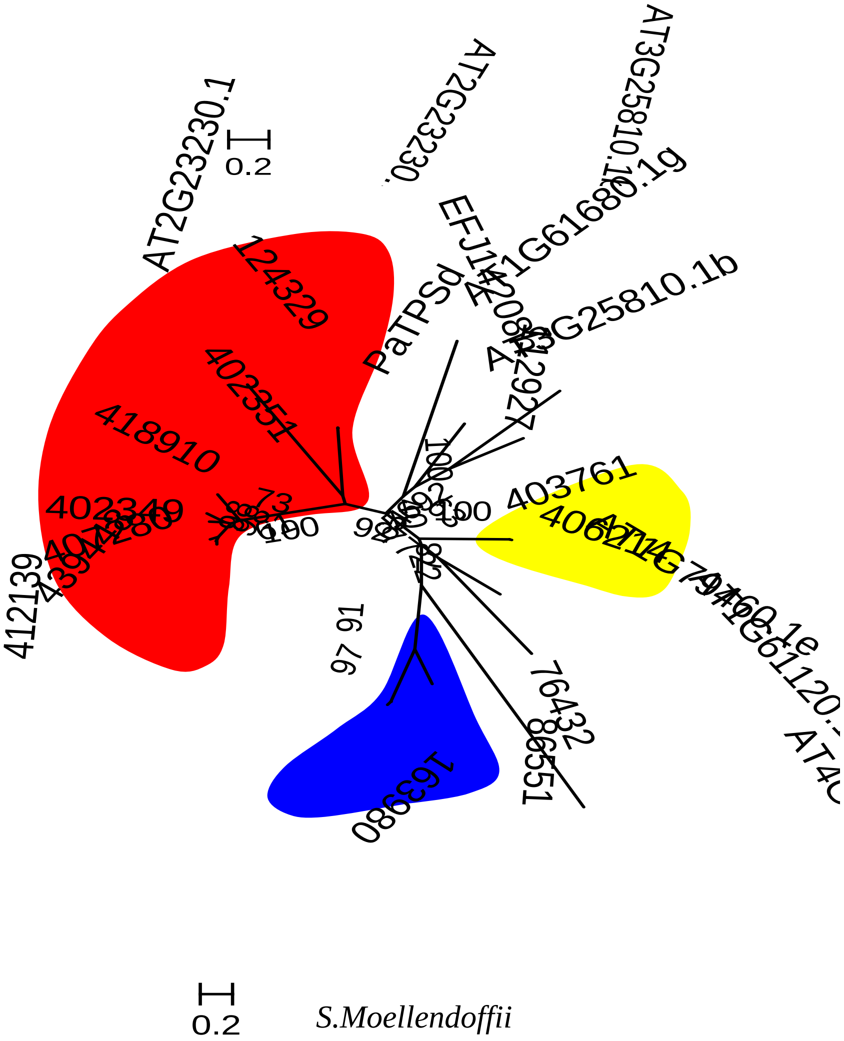

S.Moellendoffii

Supplementary Figure S1

D

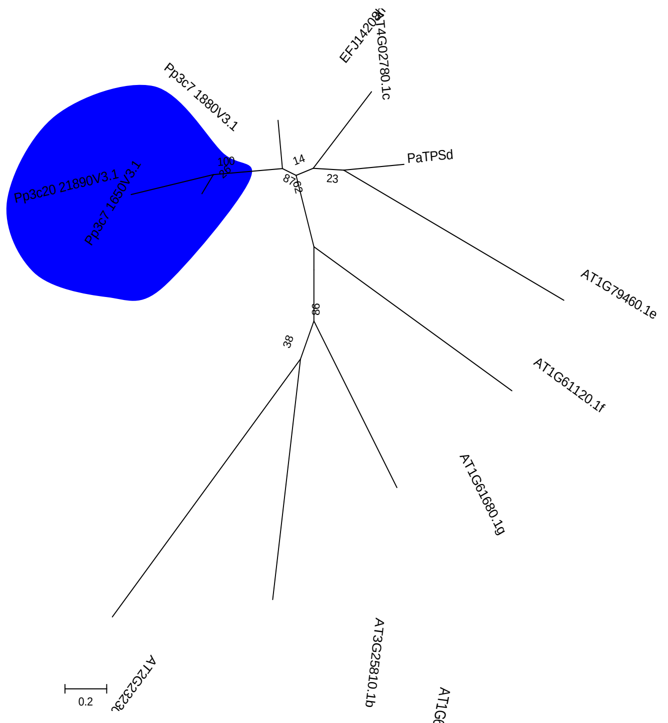

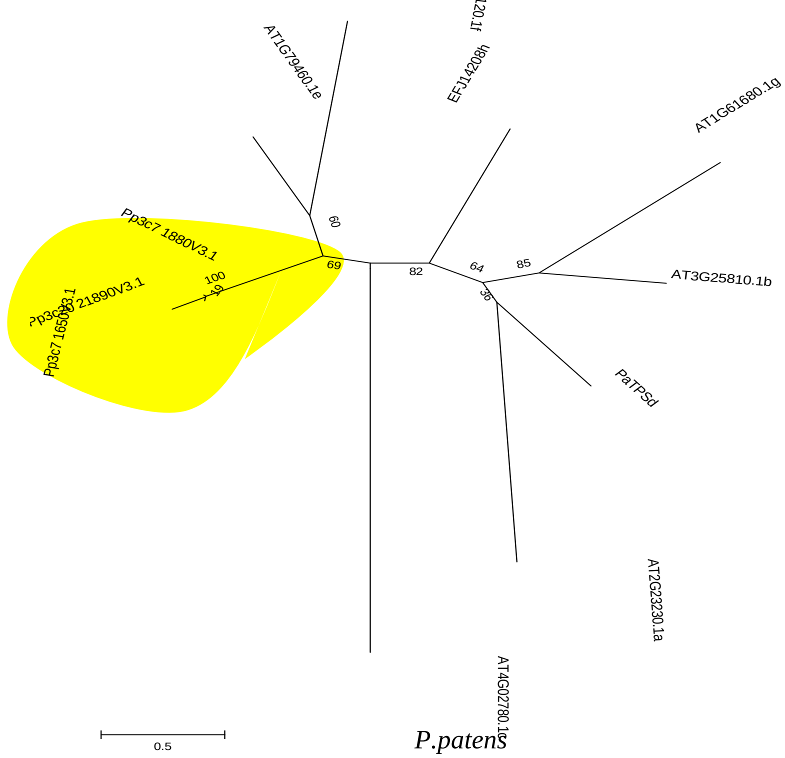

P.patens

Supplementary Figure S1. Molecular phylogenetic analysis of TPSs in additional representative species were constructed by Maximumum Likelihood method in the MEGA6 program according to the description in Methods. (A) to (D) Phylogenetic trees from *O. sativa*, *P. abies*, *S. moellendoffii* and *P. patens*, respectively. Top and bottom panels in (A) to (B) showed the phylogenetic trees based on PF03936 and PF01397 domain sequences, respectively. The subfamilies highlighted by colours purple, benzo, blue, green, yellow, orange and red, indicate TPS-a, -b, -c, -d, -e/f, -g and -h, respectively. The TPSs with no highlight in (C) to (D) were representative members from different subfamilies in other species.
