# Supplementary material for: A Comprehensive Survey on the Terpene Synthase Gene Family Provides New Insight into Its Evolutionary Patterns
Source: Genome Biol Evol. 2019 Jul 15;11(8):2078–98. doi: 10.1093/gbe/evz142 (PMC6681836; doi:10.1093/gbe/evz142)
Supplement: Supplementary_Data_evz142 [file supplementary_data_evz142.zip › Supplementary Figure S2.pdf]

Supplementary Figure S2

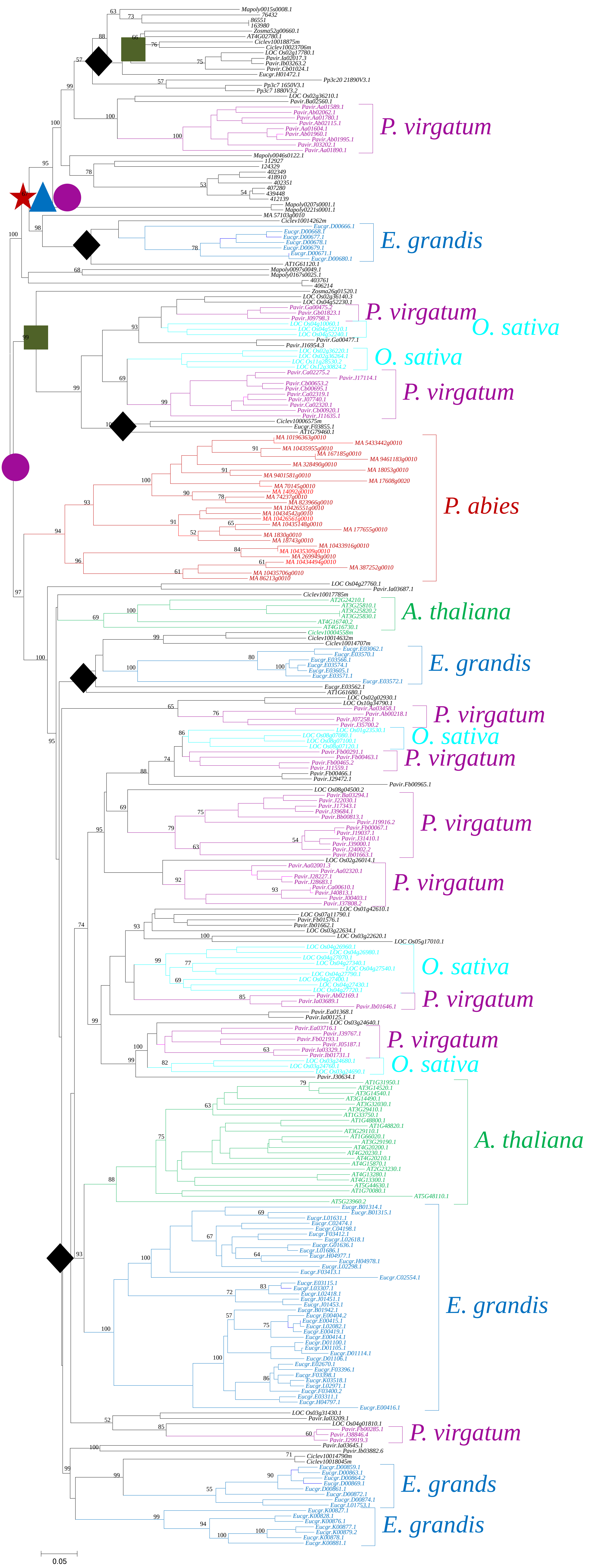

Supplementary Figure S2. The enlarged phylogenetic tree of Figure 3. The phylogenetic tree was constructed using the *TPS* members from 10 species including liverwort (*M. polymorpha*), one moss (bryophyte, *P. patens*), one lycophyte (*S. moellendorffii*), one gymnosperm (*P. abies*), three monocots (*O. sativa*, *P. virgatum*, and *Z. marina*) and three dicots (*A. thaliana*, *C. clementina* and *E. grandis*). PF01397 domain amino acid sequences were employed for the tree construction using the bootstrap method with a heuristic search in the MEGA6 program. The Bayesian analyses showed a similar result. Ancestral units were defined according to the reference (Shiu et al., Plant Cell. 2004; 16: 1220-1234). The red star represents the MRCA among all 10 organisms and the blue triangle indicates the MRCA among moss, gymnosperm and angiosperm. The pink circles show the MRCA units of gymnosperm and angiosperm. Blue squares and black diamond symbols represent the MRCA units in monocots and dicots, respectively.
