# Supplementary material for: A Comprehensive Survey on the Terpene Synthase Gene Family Provides New Insight into Its Evolutionary Patterns
Source: Genome Biol Evol. 2019 Jul 15;11(8):2078–98. doi: 10.1093/gbe/evz142 (PMC6681836; doi:10.1093/gbe/evz142)
Supplement: Supplementary_Data_evz142 [file supplementary_data_evz142.zip › Supplementary Figure S3.pdf]

Supplementary Figure S3

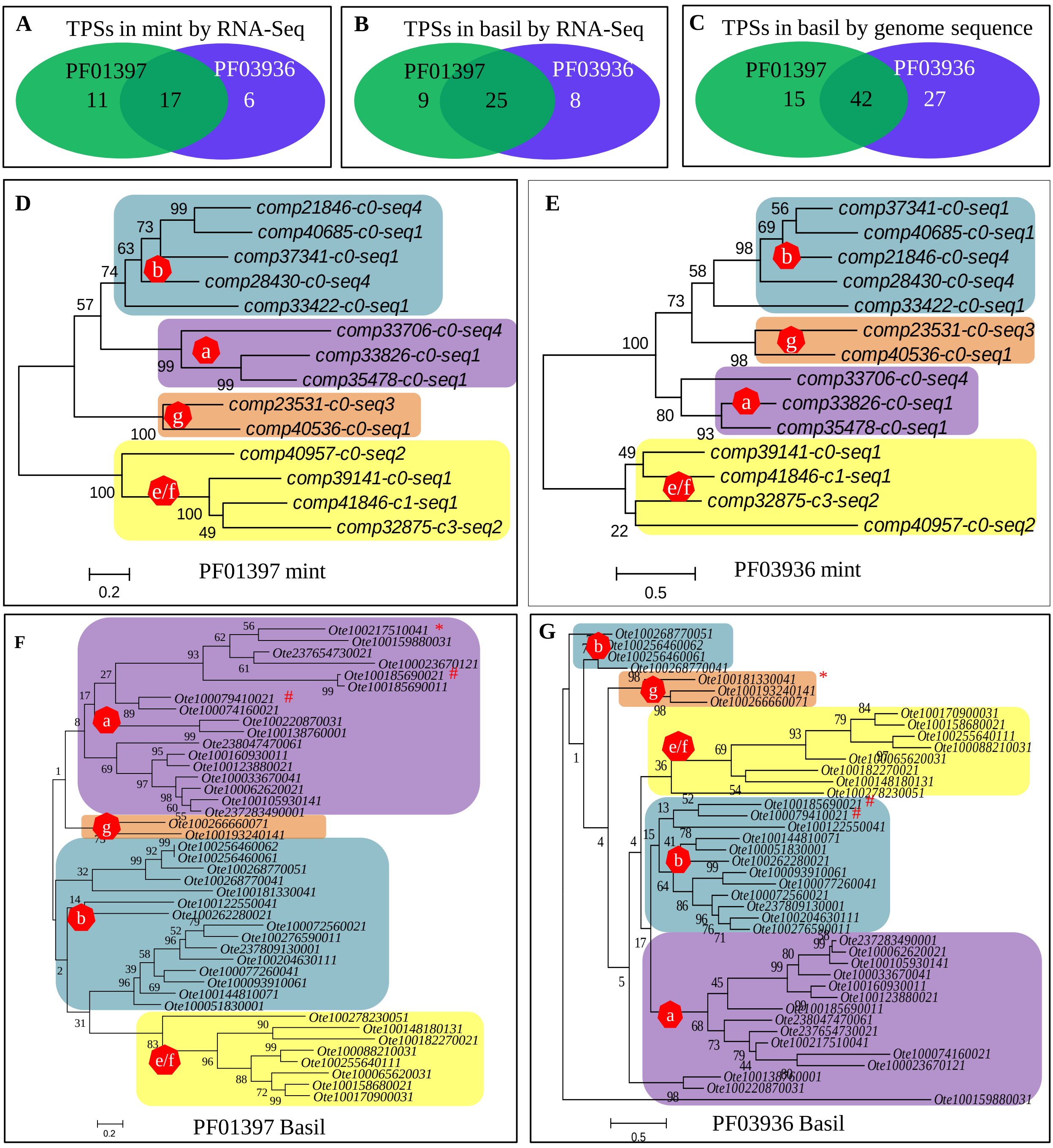

Supplementary Figure S3. Identification and characterization of the *TPS* family in mint and basil. (A) and (B) The total numbers of *TPSs* encoding the domain PF01397 or PF03936 or both of them in mint and basil, respectively, by RNA-Seq. (C) A summary of TPS members identified from the whole genome sequence database. (D) and (E) Phylogenetic analysis and classification of *TPSs* in mint based on the domain PF01397 (D) and PF03936 (e) sequences, respectively. (F) and (G) Phylogenetic analysis and classification of *TPSs* in basil based on the domain PF01397 (F) and PF03936 (G) sequences, respectively. The "*" indicated that the gene was classified into either *TPS-a* in (F) or *TPS-g* in (G). The "#" indicated that these two genes were classified into either *TPS-a* in (F) or *TPS-b* in (G).
