# Supplementary material for: A Comprehensive Survey on the Terpene Synthase Gene Family Provides New Insight into Its Evolutionary Patterns
Source: Genome Biol Evol. 2019 Jul 15;11(8):2078–98. doi: 10.1093/gbe/evz142 (PMC6681836; doi:10.1093/gbe/evz142)
Supplement: Supplementary_Data_evz142 [file supplementary_data_evz142.zip › Supplementary Figure S4.pdf]

Supplementary Figure S4

**A** Unigenes	Root	Leaf-PGT	PGT	Unigenes	Root	Leaf-PGT	PGT
comp26858_c1			*	*comp35478_c0*			*
comp30364_c1		*		*comp37341_c0*			
comp31218_c0				*comp37875_c1*			
comp32475_c1	*			*comp39141_c0*			*
comp32875_c1				*comp40536_c0*			
comp34602_c1		*		*comp40685_c0*			*
comp35437_c0			*	*comp40957_c0*			
comp36484_c2	*			*comp41846_c1*			
comp41138_c0	*			*comp43633_c0*			
comp43257_c0			*	*comp30364_c0*		*	
comp21846_c0			*	*comp34602_c0*			
comp32875_c3			*	*comp34823_c3*			*
comp33706_c0			*	*comp39134_c1*			
comp33826_c0			*	*comp40013_c0*			
comp34279_c0			*	-5.64			11.9

**B** Unigenes	Root	Leaf-PGT	PGT	Unigenes	Root	Leaf-PGT	PGT
comp41498_c0				comp80720_c0	*		
comp66727_c0				comp80868_c1			*
comp74873_c1	*			comp81202_c0			
comp79982_c0	*			comp81488_c0			
comp80953_c0		*		comp81945_c0			*
comp87432_c0	*			comp83460_c0			
comp88864_c0			*	comp83986_c0			
comp91498_c0	*			comp85201_c2			*
comp92097_c0			*	comp85742_c0			
comp11685_c0			*	comp85850_c0		*	
comp56385_c0			*	comp86221_c0			*
comp59150_c0				comp86267_c0			
comp62106_c0			*	comp86941_c0			*
comp62444_c0			*	comp105854_c0			
comp68495_c0			*	comp35948_c0			
comp70518_c0			*	comp55003_c0			
comp72455_c0	*			comp62297_c0	*		
comp73894_c0				comp72455_c3	*		
comp75097_c0			*	comp85746_c0			
comp77230_c0				comp86459_c1		*	
comp78682_c0				comp90920_c2			

-6.64 13.93

Supplementary Figure S4. Expression profiling of *TPSs* in mint and bail based on RNA-Seq analysis. (*A*) Heat map showing the expression patterns of mint *TPSs* among three different tissues including root, leaf-PGT and PGT. (*B*) Expression patterns of basil *TPSs* in three different tissues including root, leaf-PGT and PGT. In (*A*) and (*B*), the stars "*" indicated the tissue-preferred genes. The yellow cells indicate no expression of this gene in the tissue (FPKM=0). The green and blue locus names indicated TPSs encoding either PF01397 or PF03936 domain only, respectively. The black locus names indicated that the *TPSs* encode both domains.
